# In-depth profiling of COVID-19 risk factors and preventive measures in healthcare workers

**DOI:** 10.1007/s15010-021-01672-z

**Published:** 2021-08-11

**Authors:** Paul R. Wratil, Niklas A. Schmacke, Andreas Osterman, Tobias Weinberger, Jochen Rech, Burak Karakoc, Mira Zeilberger, Julius Steffen, Tonina T. Mueller, Patricia M. Spaeth, Marcel Stern, Manuel Albanese, Hella Thun, Julia Reinbold, Benedikt Sandmeyer, Philipp Kressirer, Béatrice Grabein, Peter Falkai, Kristina Adorjan, Veit Hornung, Lars Kaderali, Matthias Klein, Oliver T. Keppler

**Affiliations:** 1 Faculty of Medicine, National Reference Center for Retroviruses, Max Von Pettenkofer Institute and Gene Center, Virology, LMU München, Munich, Germany; 2 German Center for Infection Research (DZIF), Partner site, Munich, Germany; 3 Department of Biochemistry and Gene Center, LMU München, Munich, Germany; 4 Department of Medicine I, University Hospital, LMU München, Munich, Germany; 5 DZHK (German Centre for Cardiovascular Research), Partner Site Munich Heart Alliance, Munich, Germany; 6 Department of Medicine IV, University Hospital, LMU München, Munich, Germany; 7 Department of Communication and Media, University Hospital, LMU München, Munich, Germany; 8 Institute of Emergency Medicine and Management in Medicine, University Hospital, LMU Munich, Munich, Germany; 9 Department for Clinical Microbiology and Hospital Hygiene, University Hospital, LMU München, Munich, Germany; 10 Department of Psychiatry and Psychotherapy, University Hospital, LMU München, Munich, Germany; 11 Institute of Bioinformatics, University Medicine Greifswald, Greifswald, Germany; 12 Emergency Department and Department of Neurology, University Hospital, LMU München, Munich, Germany

**Keywords:** SARS-CoV-2, COVID-19, Healthcare workers, Risk factors, Prevention

## Abstract

**Purpose:**

To determine risk factors for coronavirus disease 2019 (COVID-19) in healthcare workers (HCWs), characterize symptoms, and evaluate preventive measures against SARS-CoV-2 spread in hospitals.

**Methods:**

In a cross-sectional study conducted between May 27 and August 12, 2020, after the first wave of the COVID-19 pandemic, we obtained serological, epidemiological, occupational as well as COVID-19-related data at a quaternary care, multicenter hospital in Munich, Germany.

**Results:**

7554 HCWs participated, 2.2% of whom tested positive for anti-SARS-CoV-2 antibodies. Multivariate analysis revealed increased COVID-19 risk for nurses (3.1% seropositivity, 95% CI 2.5–3.9%, *p *= 0.012), staff working on COVID-19 units (4.6% seropositivity, 95% CI 3.2–6.5%, *p* = 0.032), males (2.4% seropositivity, 95% CI 1.8–3.2%, *p* = 0.019), and HCWs reporting high-risk exposures to infected patients (5.5% seropositivity, 95% CI 4.0–7.5%, *p* = 0.0022) or outside of work (12.0% seropositivity, 95% CI 8.0–17.4%, *p* < 0.0001). Smoking was a protective factor (1.1% seropositivity, 95% CI 0.7–1.8% *p* = 0.00018) and the symptom taste disorder was strongly associated with COVID-19 (29.8% seropositivity, 95% CI 24.3–35.8%, *p* < 0.0001). An unbiased decision tree identified subgroups with different risk profiles. Working from home as a preventive measure did not protect against SARS-CoV-2 infection. A PCR-testing strategy focused on symptoms and high-risk exposures detected all larger COVID-19 outbreaks.

**Conclusion:**

Awareness of the identified COVID-19 risk factors and successful surveillance strategies are key to protecting HCWs against SARS-CoV-2, especially in settings with limited vaccination capacities or reduced vaccine efficacy.

**Supplementary Information:**

The online version contains supplementary material available at 10.1007/s15010-021-01672-z.

## Introduction

The coronavirus disease 2019 (COVID-19) caused by the severe acute respiratory syndrome coronavirus 2 (SARS-CoV-2) rapidly evolved to a pandemic in early 2020 with more than 173.4 million confirmed cases and 3.73 million deaths by June 7th, 2021 [1]. Effective treatment options for COVID-19 have not been discovered and vaccination programs are not yet available at scale in many countries, potentially weakened by the emergence of variants of concern (VOCs) [2, 3], or not well-accepted by parts of the population [4]. To this date, COVID-19 remains a major threat to global health and continues to dictate policymaking around the world.

With 5–20% of confirmed COVID-19 cases being hospitalized [5, 6], and approximately 20% subsequently requiring intensive care [7], uncontrolled SARS-CoV-2 transmission threatens to overwhelm healthcare systems [8, 9]. Ensuring adaptable and adequate hospital capacities depends heavily on the availability of skilled healthcare workers (HCWs). Given that frontline HCWs are particularly at risk of infection due to their increased exposure to SARS-CoV-2, protecting them appropriately is of high priority. Indeed, several reports of larger COVID-19 outbreaks within hospitals highlight the threat that nosocomial infections pose to both patients and HCWs [10–14]. The importance of identifying HCW-specific risk factors is underscored by the recent emergence of SARS-CoV-2 VOCs with substantially increased transmissibility, possibly elevated case fatality rates, and reduced vaccine efficacy for some [2–4, 15, 16].

Here, we report the findings from a cross-sectional study assessing SARS-CoV-2 seroprevalence as an indicator of COVID-19 in HCWs at a multicenter, quaternary care hospital in Munich, Germany. Using a questionnaire covering epidemiological and COVID-19-specific items, we identified risk groups and risk factors, characterized symptoms of SARS-CoV-2 infection, and evaluated measures to identify and prevent SARS-CoV-2 infections among employees.

## Materials and methods

### Study design, setting and participants

Between May 27th and August 12th, 2020, we invited all 11,580 employees of the LMU Klinikum, a quaternary care university hospital complex with two centers in Munich, Germany, to enroll in this cross-sectional study.

### Data collection

Participants donated a blood sample to determine the seroprevalence of antibodies against SARS-CoV-2. Furthermore, they answered an online-questionnaire assessing epidemiological, occupational, and COVID-19-specific data e.g., occurrence of symptoms, self-quarantining, or high-risk exposure to SARS-CoV-2-infected individuals (Supplementary Tables 1, 2). High-risk exposure was defined according to the criteria of the European Centre for Disease Prevention and Control [17]. The occupational health office and the HR department of the LMU Klinikum provided time-resolved numbers of hospitalized COVID-19 patients, and SARS-CoV-2-infected or quarantined HCWs, respectively.

### Anti-SARS-CoV-2 antibody detection assays

The following four commercial tests were used according to the manufacturers’ instructions to determine the presence of SARS-CoV-2-specific antibodies in serum specimens: Architect SARS-CoV-2 IgG (Abbott, Illinois, USA), Anti-SARS-CoV-2-ELISA IgG (EuroImmun, Lübeck, Germany), Elecsys^®^ Anti-SARS-CoV-2 (Roche, Basel, Switzerland), and recomLine SARS-CoV-2 IgG (Mikrogen, Neuried, Germany). We included a threshold for indeterminate test results in the Elecsys^®^ assay at 0.8 COI value. Additionally, a self-developed assay was utilized. Herein, 96-well high-binding plates were coated overnight at 4 °C with purified, trimeric SARS-CoV-2 spike protein (1 µg/mL, 50 µL/well) in 0.1 M sodium carbonate pH = 9.57, and blocked with 3% milk in 0.05% Tween-20 in PBS (PBST, 100 µL/well) for 1 h at RT. After blocking, plates were incubated for 1 h at RT with 50 µL/well heat-inactivated patient serum samples diluted 1:150 in PBS containing 1% milk. Subsequently, horseradish peroxidase (HRP) conjugated goat anti-human IgG antibody (Sigma-Aldrich A0293, 50 µL/well, diluted 1:3000 in 1% milk in PBST) was added and samples were incubated for 1 h at RT. After all steps mentioned above, plates were washed with PBST. For the HRP-catalyzed reaction, samples were incubated with 50 µL/well BD OptEIA^™^ TMB substrate (BD Biosciences, New Jeresey, USA) and the reaction was stopped after 10 min by addition of 50 µL/well 5% H_2_SO_4_. Finally, absorption was recorded at 450 nm. Samples were called indeterminate or positive with a background-subtracted absorption of more than 15% (indeterminate) and 45% (positive) of the absorption of a uniform plate-wise positive control that consisted of several pooled sera from hospitalized COVID-19 patients.

The performance of the anti-SARS-CoV-2 antibody detection assays was determined on a set of 1152 pre-pandemic serum samples from adults and children, as well as 332 specimens from 99 COVID-19 patients (Supplementary Tables 3, 4).

Sera from all participants were tested using both the Elecsys^®^ assay, and the self-developed ELISA. Samples that were tested negative in both screening assays, but either scored indeterminate in at least one of the two assays or originated from a participant who reported a positive SARS-CoV-2 rRT-PCR result in the study questionnaire, were further analyzed via the other assays (Supplementary Fig. 1a). As COVID-19 vaccines were not administered to HCWs at the LMU Klinikum before or during study sampling, the detection of anti-SARS-CoV-2 antibodies in participants’ sera was indicative of (sub-)acute or resolved SARS-CoV-2 infection and therefore, according to the case definition of the European Centre for Disease Prevention and Control (ECDC), these HCWs were classified as COVID-19 cases [18].

### SARS-CoV-2 neutralization assay

CaCo-2 cells (American Type Culture Collection, ATCC, Virginia, USA) in cell culture medium (Dulbecco’s Modified Eagle’s Medium containing 2% fetal bovine serum) were challenged for 2 h with a clinical isolate (GISAID EPI ISL 4,66,888) previously obtained from a nasopharyngeal swab of a COVID-19 patient. Subsequently, cell culture medium was exchanged, and three days post infection supernatants were passaged on Vero-E6 cells (ATCC). After three additional days, cell culture supernatants were harvested and stored at −80 °C. The virus stock was characterized by rRT-PCR and by titration on human lung epithelial A549 cells (ATCC), overexpressing the human angiotensin-converting enzyme 2 receptor, ACE2 (A549-hACE2 cells).

A volume of this virus stock, which results in a 90% cytopathic effect three days post infection, was incubated for 2 h with patient sera at different dilutions. Subsequently, 10 µL of the virus-serum mixtures were added to 20 µL A549-hACE2 cells cultured in 384-well plates (7500 cells/well). Three days post infection, 10 µL of CellTiter-Glo^®^ 2.0 reagent (Promega, Wisconsin, USA) were added to each well and the luminescence recorded (0.5 s integration time, no filter). The half-maximal inhibitory concentrations (IC_50_) for inhibiting virus-mediated cell death were computed via normalized sigmoidal dose–response curve approximation with variable slopes. Neutralizing activities were categorized via the obtained IC_50_ values: none (IC_50_ < tenfold serum dilution), low (IC_50_ < 90-fold serum dilution), intermediate (IC_50_ < 270-fold serum dilution), high (IC_50_ < 2430-fold serum dilution), very high (IC_50_ ≥ 2430-fold serum dilution).

### Statistical analysis

Data were analyzed in R version 4.0.3 (www.r-project.org) using the R package epitools. Parameters of multivariate significance are the result of a logistic regression, using recursive elimination of the least significant remaining factor. *p* values on pair-wise comparisons were calculated using Fisher’s exact test with Holm’s multiple testing correction as indicated. Decision trees were computed using the party package in R with default parameters [19]. Confidence intervals for absolute risks were calculated with Wilson’s method using the binconf function from the Hmisc R package.

## Results

### Pandemic situation and study population

**Fig. 1:**
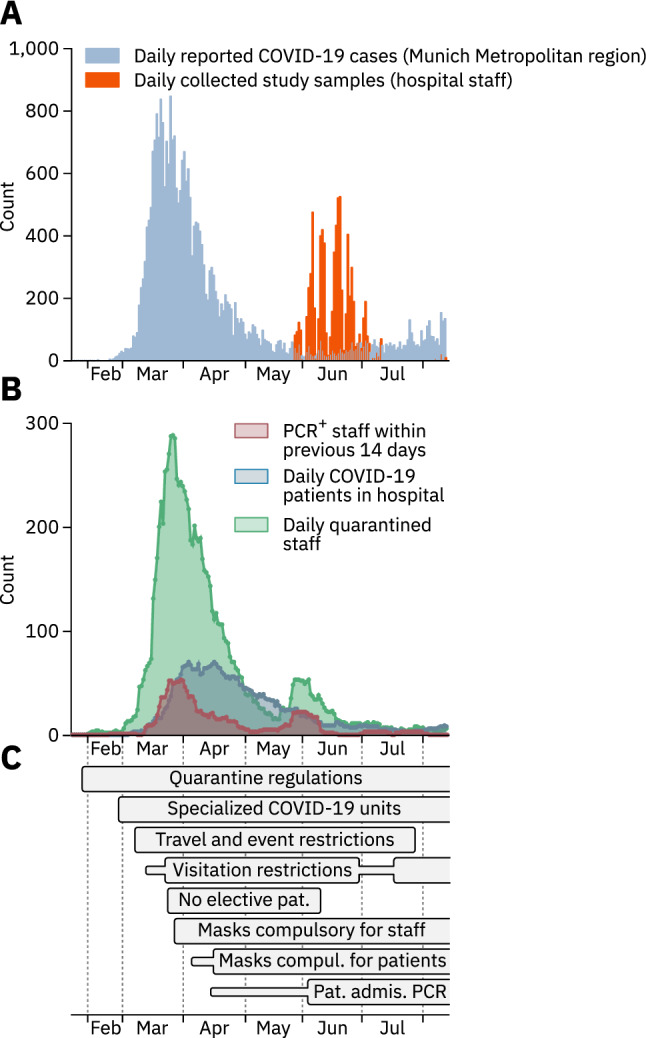
Dynamics of the COVID-19 pandemic and implementation of preventive measures. **a** COVID-19 cases officially reported for the Munich metropolitan region until August 12th, 2020 (blue) and the number of blood samples collected from staff members (orange) are depicted as one bar per day. **b** Number of HCWs who tested positive for SARS-CoV-2 by PCR within a two-week window preceding the reported date (red), number of COVID-19 patients treated in the hospital (blue), and number of hospital staff in quarantine (green). **c** Time-resolved depiction of state-imposed and institutional measures taken to prevent SARS-CoV-2 spread at the multicenter hospital. Thinner, horizontal bars represent less strict measures of the same type. Measures that were still in effect by August 12th, 2020 are depicted as bars with open endings. Pat. Admis. PCR – Mandatory PCR test for newly admitted patients

Until August 12th, 2020, the Munich Metropolitan region was among the areas most severely affected by the COVID-19 pandemic in Germany (Fig. 1a, blue), accounting for 12.8% (28,010/2,18,519) of all cases registered [20]. Quarantining (Fig. 1b, green) was mandatory for SARS-CoV-2 PCR-positive HCWs (Fig. 1b, red), those who returned from designated high-risk areas [21], and for HCWs non-essential for patient care reporting high-risk exposures to infected individuals. Until August 12th, 2020, 231 COVID-19 patients were hospitalized at the quaternary care hospital surveyed here, at peak times 70 per day (Fig. 1b, blue), and several COVID-19 countermeasures were implemented (Fig. 1c, Supplementary Table 5).

Between May 27th and August 12th, 2020, after the first wave of the COVID-19 pandemic had largely subsided, we invited all 11,580 staff members of the multicenter hospital to submit a blood sample for analysis of anti-SARS-CoV-2 antibodies (Fig. 1a, orange), and to complete a questionnaire. 7554 employees (65.2% of all staff) participated, 2.2% (166/7554) of whom tested positive for anti-SARS-CoV-2 antibodies (Supplementary Fig. 1a). Results from the two screening assays agreed in 98.1% (7349/7491) of cases (Supplementary Fig. 1b). Seropositivity was most frequent among HCWs under 30 years of age (2.95%, Table 1). More participants were female (5431/7553, 71.9%), and male gender was a COVID-19 risk factor in multivariate analysis (2.41% seropositivity, 95% CI 1.8–3.2, *p* value for multivariate analysis (*p*_*m*_) = 0.019, Table 2). 88.2% (164/186) of serum samples from anti-SARS-CoV-2 antibody positive (Ab^+^) HCWs or those reporting positive SARS-CoV-2 PCR results exhibited neutralizing activity (Supplementary Fig. 2a, b). This neutralizing activity correlated with antibody titers, but not with the time elapsed since a positive PCR test (Supplementary Fig. 2c, d).

**Table 1:** Epidemiological information and anti-SARS-CoV-2 antibody status of 7554 healthcare workers participating in the study

	Anti-SARS-CoV-2 Ab		95% CI
	Positive/total	%	
Total	166/7554	2.20	1.89–2.55
Age group (*Y*)			
≤ 30	64/2170	2.95	2.32–3.75
31–40	39/1951	2.00	1.47–2.72
41–50	29/1430	2.03	1.42–2.90
51–60	23/1467	1.57	1.05–2.34
> 60	11/536	2.05	1.15–3.64
Gender			
Female	115/5431	2.12	1.77–2.54
Male	51/2118	2.41	1.84–3.15
3rd gender	0/5	0.00	
Patient care occupations			
Nurse	68/2185	3.11	2.46–3.93
Physician	38/1345	2.83	2.07–3.85
Other	17/1199	1.42	0.88–2.26
Total	123/4729	2.60	2.18–3.10
Non-patient care occupations			
Administration/IT	15/822	1.82	1.11–2.99
Research	12/977	1.23	0.70–2.14
Transportation	1/28	3.57	0.63–17.71
Cleaning personnel	4/119	3.36	1.32–8.33
Other	11/879	1.25	0.70–2.23
Total	43/2825	1.52	1.13–2.04

Binominal 95% confidence intervals (95% CI) were calculated using the Wilson score interval

**Table 2:** Significant risk and protective factors for SARS-CoV-2 seropositivity among participants in multivariate analysis

Parameter	Anti-SARS-CoV-2 Ab			*p*_*m*_ value	*Z* value
	Positive/total	%	95% CI		
All participants	166/7554	2.2	1.9−2.6		
Male gender	51/2067	2.4	1.8–3.2	0.019	2.35
Active smoking behavior	16/1407	1.1	0.7–1.8	0.00018	−3.74
Works in non-clinical department	9/1149	0.8	0.4–1.6	0.017	−2.55
Working on COVID-19 unit	28/583	4.6	3.2–6.5	0.032	2.14
High-risk exposure to infected patients	38/651	5.5	4.0–7.5	0.0022	3.06
High-risk exposure in community	22/162	12.0	8.0–17.4	< 0.0001	5.04
Occupation: nurse	68/2117	3.1	2.5–3.9	0.012	2.52
Symptom: taste disorder	72/170	29.8	24.3–35.8	< 0.0001	14.81
Symptom: sore throat	53/1853	2.8	2.1–3.6	< 0.0001	−4.35
Symptom: fatigue	86/1413	5.7	4.7–7.0	< 0.0001	4.76
Patient contacts primarily in operating theaters	9/896	1.0	0.5–1.9	< 0.0001	−4.06

Binominal 95% confidence intervals (95% CI) were calculated using the Wilson score interval

Logistic regression followed by recursive feature elimination up to a threshold of *p* = 0.05. *p*_*m*_ value−*p *value for multivariate analysis

### High-risk exposure to infected individuals

**Fig. 2:**
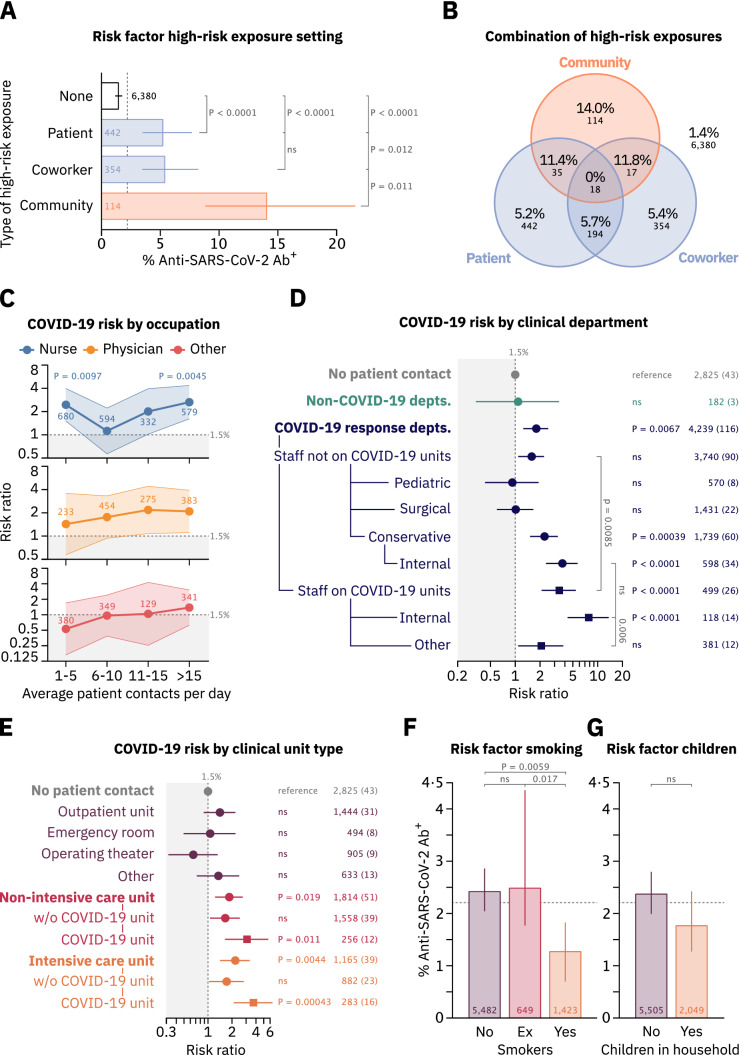
Risk factors for SARS-CoV-2 seropositivity among healthcare workers. **a** Percentage of SARS-CoV-2 seropositive HCWs by self-reported instances of different types of high-risk exposure. Only staff reporting exposures of a single type is shown. **b** Percentages and absolute numbers of SARS-CoV-2 Ab^+^ staff members self-reporting combinations of high-risk exposures in different settings. Numbers outside the diagram correspond to staff members in none of the depicted groups. **c** SARS-CoV-2 seropositivity risk ratio (RR) of nurses, physicians and other patient-facing HCWs and average self-reported patient contacts per day relative to staff without patient contact (RR set to 1). Shaded areas depict 95% confidence intervals (CIs). *p* values from Fisher’s exact test are reported where *p* < 0.05. **d** SARS-CoV-2 seropositivity RRs for HCWs originating from different departments relative to staff without patient contact (RR set to 1). Departments that deployed staff members to COVID-19 units are termed “COVID-19 response depts.”, all others are grouped under “non-COVID-19 depts.”. Staff from COVID-19 response departments were further stratified according to their deployment to COVID-19 units and to the medical specialty of their department. Dots represent risk ratios, while lines indicate 95% CIs. **e** SARS-CoV-2 seropositivity RRs for HCWs self-reporting patient contact on different types of clinical units. Multiple selections were possible. **f** Self-reported smoking behavior and risk for SARS-CoV-2 seropositivity. Bars represent percentages of anti-SARS-CoV-2 Ab^+^ staff. Error bars represent 95% CIs. **g** Self-reported number of children living in the same household with HCWs as a risk factor for SARS-CoV-2 seropositivity. *p* values in **a**, **d**–**g** were calculated using Fisher’s exact test and are reported as adjusted *p* values after Holm’s multiple testing correction. Numbers next to datapoints indicate number of staff members per group and numbers in braces indicate number of Ab^+^ staff members (**c**–**e**). Dotted lines correspond to the risk of staff without patient contact (**c**–**e**, 1.5%) or number of SARS-CoV-2 Ab^+^ staff from the entire dataset (**a**, **f**, **g**, 2.2%)

Participants were asked to report high-risk exposures (defined according to the criteria of the ECDC [17]) to either patients, co-workers, or individuals in their non-work-related environment (“community”) with acute COVID-19. High-risk exposures within a HCW’s community or to COVID-19 patients were risk factors for SARS-CoV-2 infection in multivariate analysis (12.0% seropositivity, 95% CI 8.0–17.4, *p*_*m*_ < 0.0001, and 5.5% seropositivity, 95% CI 4.0–7.5, *p*_*m*_ = 0.0022) (Table 2). Moreover, compared to staff members without high-risk exposure, HCWs’ exposures in the hospital to either infected co-workers (risk ratio (RR) 3.76, 95% CI 2.32–6.10) or COVID-19 patients (RR 3.65, 95% CI 2.33–5.71), and especially to infected individuals in the community (RR 9.84, 95% CI 5.98–16.19) resulted in increased risk for seropositivity (*p* < 0.0001 for all three comparisons) (Fig. 2a, Supplementary Fig. 3a). Dual high-risk exposures to either co-workers or patients in combination with an exposure in the community led to greater COVID-19 risk than exposures in the hospital alone (Fig. 2b). However, markedly more HCWs reported high-risk exposures in the hospital than in their community (Fig. 2a). 55% (91/166) of seropositive HCWs did not report any high-risk exposure, underscoring the importance of unrecognized exposure for infection.

### Occupation-specific risk factors

Nurses, doctors, cleaning- and transport personnel had the highest risk for seropositivity (Table 1) and working as a nurse was a risk factor of multivariate significance (3.1% seropositivity, 95% CI 2.5–3.9, *p*_*m*_ = 0.011, Table 2). HCWs with low risk included researchers and medical technicians. Generally, patient-facing HCWs were more at risk for SARS-CoV-2 infection than non-patient-facing HCWs (RR 1.77, 95% CI 1.25–2.50, *p* = 0.002, Table 1). Frequent patient contacts increased the COVID-19 risk across all patient-facing occupations (Fig. 2c). Nurses reporting six to ten patient contacts per day had a noticeably low risk (Fig. 2c, blue line). 36.7% (218/594) of nurses in this group worked in operating theaters (Supplementary Fig. 4a), where few COVID-19 patients were treated, and nurses’ overall risk was lowest (Supplementary Fig. 4b). Nurses reporting between one and five patient contacts per day were, in turn, highly at risk for SARS-CoV-2 infection. Analysis of this subgroup revealed that 75.1% (511/680) worked on intensive care units (ICUs, Supplementary Fig. 4a), where, despite few patient contacts, nurses were highly at risk (Supplementary Fig. 4b).

### Department- and unit-specific risk factors

The majority of departments deployed staff members to COVID-19 units (Supplementary Table 6). Among HCWs from these “COVID-19 response departments” who did not work on COVID-19 units, only personnel from conservative departments showed an increased rate of seropositivity compared to personnel without patient contact (RR 2.27, 95% CI 1.54–3.34, *p* = 0.0004). Within this group, HCWs in departments of internal medicine had a markedly increased COVID-19 risk (RR 3.74, 95% CI 2.40–5.81, *p* < 0.0001, Fig. 2d). Working on COVID-19 units was associated with an overall increased risk for seropositivity in a multivariate model (4.6% seropositivity; 95% CI 3.2–6.5, *p*_*m*_ = 0.032, Table 2). Among personnel working on COVID-19 units, staff members from internal medicine departments were highly at risk compared to non-patient-facing HCWs (RR 7.80, 95% CI 4.39–13.84, *p* < 0.0001), and even compared to employees on COVID-19 units from other departments (RR 3.47, 95% CI 1.65–7.32, *p* = 0.006, Fig. 2d). Staff working in non-clinical departments, including those without patient contact, had a significantly decreased risk for SARS-CoV-2 infection in a multivariate model (0.78% seropositivity, 95% CI 0.41–1.46, *p*_*m*_ = 0.0179, Table 2).

Regarding COVID-19 risk in relation to patient contacts on different types of clinical units, HCWs both on ICUs and non-ICUs treating COVID-19 patients had an increased risk (RR 3.08, 95% CI 1.65–5.76, *p* = 0.011, and RR 3.71, 95% CI 2.12–6.51, *p* = 0.00043), whereas HCWs in outpatient units, operating theaters, and in the emergency room (ER) had a largely unaltered risk compared to non-patient-facing employees (Fig. 2e). Notably, of the 28 Ab^+^ staff members working on COVID-19 units, none reported high-risk exposures in the community, while 18 (64.3%) reported high-risk exposures in the hospital (Supplementary Fig. 5a). There were no significant differences in the risks for SARS-CoV-2 infection for HCWs being deployed to COVID-19 units or those not working on COVID-19 units comparing employees from the two different study centers i.e., Central Munich and Großhadern (Supplementary Fig. 5b).

### Smoking behavior, children in household and medical preconditions

Interestingly, self-reported smoking behavior was associated with decreased COVID-19 risk compared to non-smokers (RR 0.47, 95% CI 0.28–0.78, *p* = 0.0059) or employees that stopped smoking within the last ten years (ex-smoker, RR 0.41, 95% CI 0.21–0.79, *p* = 0.017) (Fig. 2f) and in multivariate analysis (*p*_*m*_ = 0.00018, Table 2). HCWs with children in their households and those reporting medical preconditions were not at increased risk for SARS-CoV-2 infection (Fig. 2g, Supplementary Fig. 5c). Of note, schools and kindergartens in the area were closed between March 16th and May 11th, 2020.

### Symptoms

**Fig. 3:**
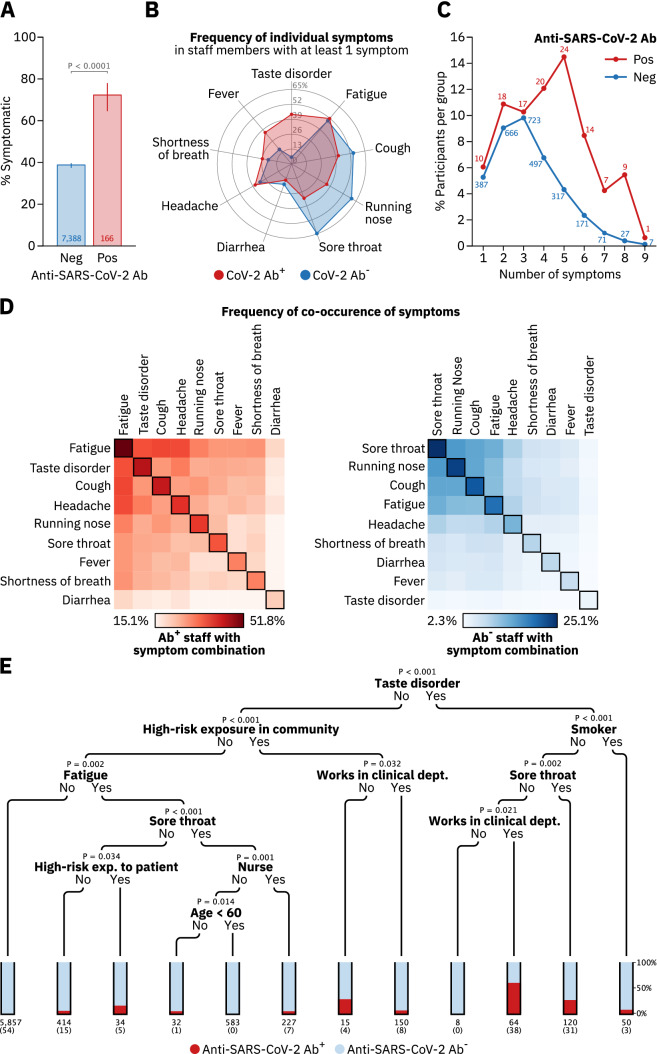
COVID-19 associated symptoms in healthcare workers and risk stratification in an unbiased decision tree. **a** Percentage of SARS-CoV-2 Ab^+^ and Ab^−^ HCWs who reported having experienced at least one of nine symptoms shown in **b**. *P*-value was calculated using Fisher’s exact test. **b** Frequency of individual symptoms in SARS-CoV-2 Ab^+^ and Ab^−^ staff members with at least one self-reported symptom as a percentage of the respective group. **c** Percentage of SARS-CoV-2 Ab^+^- and Ab^−^ staff reporting the indicated number of symptoms. Numbers beside data points indicate number of staff members per group. **d** Frequency of co-occurrence of pairs of symptoms in Ab^+^ (red) and Ab^−^ (blue) staff members. Squares on the diagonal represent the frequency of single symptoms. **e** A conditional inference tree (decision tree) was trained in R using the *ctree* function implemented in the party package, using default parameters. All significant parameters from the logistic regression were included in the training dataset. Depicted is the resulting decision tree with the stop-criterium for tree splits set at a significance level of *α* = 0.05. Numbers underneath bars represent the total number of HCWs in the respective group, numbers in braces those of Ab^+^ staff members

HCWs were asked to report symptoms they had experienced within the previous three months. 72.2% (120/166) of Ab^+^ HCWs noted at least one of nine symptoms given, while 27.7% (46/166) were asymptomatic (Fig. 3a). Taste disorder was the symptom with the highest predictive value for SARS-CoV-2 infection (*p*_*m*_ < 0.0001, Table 2, with 43.4% (72/120) of symptomatic Ab^+^ HCWs experiencing taste disorder compared to only 5.9% (170/2866) of symptomatic anti-SARS-CoV-2 antibody negative (Ab^−^) HCWs (Fig. 3b). Cold-like symptoms, such as sore throat, running nose or cough, in contrast, had low predictive value for COVID-19, sometimes even being more frequent among Ab^**−**^ HCWs (Fig. 3b). Overall, symptomatic Ab^+^ staff members experienced more symptoms compared to their symptomatic Ab^**−**^ counterparts (Fig. 3c). No symptom combination provided a predictive signature for COVID-19 in HCWs (Fig. 3d). The most specific symptom complex for COVID-19 was taste disorder, headache, fatigue and fever, with 46.9% (23/49) of all HCWs reporting this complex being Ab^+^ (Supplementary Fig. 6a). However, this combination of symptoms was reported by only 13.9% (23/166) of all Ab^+^ HCWs.

### Risk stratification in an unbiased decision tree

We built a decision tree based on all parameters with multivariate significance (Table 2) to identify classifiers for high- and low-risk subgroups among HCWs (Fig. 3e). Nodes in the tree represent the parameters that most significantly bisect the respective subgroup of HCWs into seropositive and negative. For example, of these classifying parameters, high-risk exposures in the community most significantly identified seropositive HCWs in the subgroup of those who did not experience taste disorder. Taste disorder had the highest predictive value for seropositivity on the entire dataset and smoking as well as working as a nurse were strong predictors of an Ab^**−**^ or Ab^+^ outcome in the indicated subgroups, respectively. Interestingly, working in a clinical department can significantly identify both a higher and a lower-risk population in different subgroups. Having a sore throat predicted a lower COVID-19 risk in two separate subgroups (Fig. 3e).

### Quarantining and working from home

**Fig. 4:**
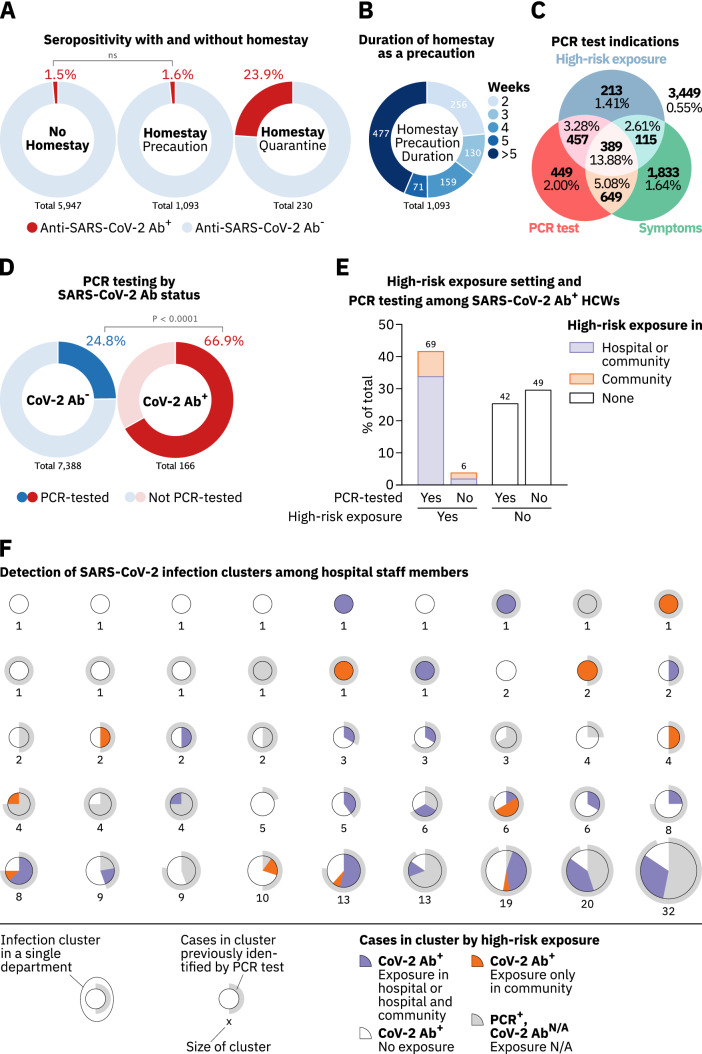
Effectiveness of measures to track and prevent SARS-CoV-2 transmission in hospital staff. **a** SARS-CoV-2 serostatus among staff reporting to have stayed at home for at least two weeks either as a precaution (middle circle) or quarantined (right circle) in comparison to staff members not staying at home (left circle). Participants who indicated to have been quarantined or stayed at home for at least two weeks without reporting to have worked from home were considered quarantined. **b** HCWs who stayed home as a precaution for at least two weeks grouped by the duration of their homestay. **c** Total numbers and percentages of anti-SARS-CoV-2 Ab^+^ HCWs who self-reported on (1) having been tested by PCR, (2) experienced at least one symptom depicted in Fig. 3B, or (3) had a high-risk exposure. **d** Numbers and percentages of anti-SARS-CoV-2 Ab^+^ and Ab^−^ staff who were tested for SARS-CoV-2 infection by PCR. **e** Percentages of anti-SARS-CoV-2 Ab^+^ HCWs who were tested for SARS-CoV-2 infection by PCR or reported a high-risk exposure in (1) the hospital or the hospital and their community (blue) or (2) their community only (orange). **f** Analysis of SARS-CoV-2 infection clusters and their detection among HCWs in the hospital. Each pie chart represents one infection cluster and clusters are separated by departments. Inner pie charts represent high-risk exposure types reported by Ab^+^ study participants in each cluster (blue, orange and white). Grey areas in inner pie charts represent individuals who were PCR-tested at the hospital but did not participate in this study. Grey circles around each pie chart represent the cluster’s fraction of COVID-19 cases previously identified by PCR testing. Numbers below the pie charts indicate the amount of SARS-CoV-2-infected HCWs in each cluster. Study participants reporting a positive PCR test in the study questionnaire were assumed to be identical to those registered at the occupational health office. HCWs who were PCR-tested at the hospital complex but did not participate in the study were added to the respective clusters as recognized cases (grey areas in inner pie charts). *p* values in **a**, **e** were calculated using Fisher’s exact test

Participants were asked to report whether they self-quarantined or worked from home as a preventive measure. Since HCWs self-quarantined upon confirmed or suspected SARS-CoV-2 infection, the rate of Ab^+^ individuals in this group was high (23.9%, Fig. 4a). While working from home reduced high-risk exposures to infected co-workers, it did not reduce such exposures within the HCW’s community and, surprisingly, did not lower the overall COVID-19 risk (RR 1.06, 95% CI 0.63–1.77) (Fig. 4a, Supplementary Fig. 7a, b), despite 76.6% (837/1093) of these homestays continuing for at least three weeks (Fig. 4b). Of note, working from home as a precaution was only possible for those employees whose presence in the hospital was not essential for patient care.

### Evaluation of the PCR-testing strategy

Major indications for SARS-CoV-2 testing by PCR in HCWs were presentation with COVID-19-associated symptoms and reporting high-risk exposures. The seropositivity rate among the group who reported neither testing indication nor having been PCR-tested was four-fold lower (0.55%) than the average seropositivity rate observed in this study (2.20%, Fig. 4c). 72.1% (846/1174) of HCWs who reported a high-risk exposure in the questionnaire were also tested by PCR. Of the remaining 27.9% (328/1174), 64.9% (213/328) were asymptomatic. Among staff members reporting high-risk exposures in the hospital that were not tested by PCR, 66.5% (189/284) reported not having notified the occupational health office about this perceived risk, despite being obligated to do so. Overall, 75.8% (964/1272) of all high-risk exposures to SARS-CoV-2-infected individuals in the hospital (to patients or co-workers) were reported to the occupational health office, with no difference between occupations (Supplementary Fig. 7c).

34.8% (1038/2986) of all staff members reporting at least one symptom were tested by PCR, and symptomatic HCWs who were tested by PCR were more likely to seroconvert compared to non-PCR-tested, symptomatic HCWs indicating that not all symptoms listed in the study questionnaire urged employees to get PCR-tested (Fig. 4c). Indeed, three of the four symptoms that constitute the symptom combination with the highest predictive value for an Ab^+^ status i.e., taste disorder, fever and headache, were more abundant among symptomatic staff members who got PCR-tested, irrespective of whether participants had additionally reported high-risk exposures to individuals with COVID-19 (Supplementary Fig. 8a). 66.9% (111/166) of Ab^+^, compared to 24.8% (1832/7388) of Ab^**−**^ HCWs, had been tested by PCR at least once (Fig. 4d). Focusing on the group of Ab^+^ participants, we found that 92.0% (69/75) of those indicating a high-risk exposure had been tested by PCR (Fig. 4e). Among Ab^+^ HCWs without high-risk exposures, 46.2% (42/91) had been PCR-tested (Fig. 4e). Of the 55 seroconverted HCWs who reported not having been tested by PCR, 40.0% (22/55) were asymptomatic.

Combining data on PCR testing of HCWs provided by the occupational health office and pseudonymized data from study participants, we investigated the occurrence of potentially unrecognized COVID-19 clusters. No cluster of more than two HCWs participating in this study remained undetected in individual organizational units (Fig. 4f). In all COVID-19 clusters among Ab^+^ HCWs involving more than 10 individuals,  ≥ 75% of the cluster size had been detected by PCR (Fig. 4f, outer grey circles), with higher rates of unrecognized cases in those clusters that also contained more HCWs who did not report any high-risk exposure (Fig. 4f, white areas in pie charts).

## Discussion

In this cross-sectional study conducted at a multicenter quaternary care hospital at the end of the first pandemic wave we identified several occupation-specific COVID-19 risk factors for HCWs, including high-risk exposures in the hospital and the community, working in patient-facing occupations, particularly as nurses, in departments of internal medicine, and on COVID-19 units, as well as being of male gender. Surprisingly, we found smoking behavior to be protective against SARS-CoV-2 infection. Among the symptoms analyzed, especially taste disorder was highly associated with COVID-19.

A common strategy to cope with hospital-associated COVID-19 is vaccinating HCWs against SARS-CoV-2. In many countries, however, vaccination programs are not yet available at scale. Furthermore, for some of the recently emerged VOCs that are spreading rapidly, reduced vaccine efficiencies have been reported [2, 3]. New VOCs escaping current vaccine responses may develop over the next months [22] resulting in an increased risk of infection at a population level irrespective of the vaccination status. Moreover, in certain countries, a considerable fraction of citizens, among them HCWs, are reluctant to become vaccinated against SARS-CoV-2 [4]. Consequently, the identification of occupation-specific risk factors in HCWs and the evaluation of surveillance strategies as well as preventive measures remain crucial to ensure adequate hospital capacities in the COVID-19 pandemic.

A study conducted in the New York Metropolitan region, USA, found no hospital-specific risk factors for SARS-CoV-2 infection in HCWs [23]. However, the overall prevalence of Ab^+^ individuals in New York State was estimated to be 6.9–14.0% by the end of April 2020 [24, 25]. In contrast, data from Munich, Germany, the city in which our study was conducted, indicate a seroprevalence of only 1.8%, by the end of April 2020 [26]. Conceivably, high prevalence concomitant with a high risk of transmission in the community may overshadow the identification of hospital-specific risk factors for HCWs. This is underscored by the relevance of high-risk exposures in the community for HCWs reported here and by others [27–30]. We hypothesize that private high-risk exposures might overall be longer and more intense than professional exposures in the hospital setting, and the former thus more contagious. Congruently, we discovered that working from home as a preventive measure did not reduce the risk of seropositivity in HCWs. However, at the hospital complex surveyed here, only those employees were eligible for working from home whose presence at the hospital was not crucial to ensure adequate patient care i.e., mainly those individuals working in non-patient-facing occupations. Whether working from home may have been protective for patient-facing HCWs, therefore, cannot be answered by our study.

The aforementioned overshadowing effect of SARS-CoV-2 transmission in the community could also explain why studies conducted in high prevalence areas did not identify working on ICUs to be associated with increased risk for seropositivity [27, 31]. We observed the contrary, especially for nurses, even though ICU nurses reported fewer patient contacts per day compared to their colleagues working on other wards.

Other studies identified, in part, similar COVID-19 risk factors in HCWs compared to ours, including male gender [32], working in patient-facing occupations [32, 33], on COVID-19 units and in departments of internal medicine [31, 32], as well as taste disorder [32]. However, several risk and protective factors described here, such as working as a nurse and high-risk exposure in the hospital were thus far unknown. Moreover, we show in this study for the first time that certain COVID-19 risk factors among HCWs are statistically significant in multivariate analysis, thus underlining their importance.

High-risk exposures in hospitals can be minimized by strictly enforcing patients and staff to wear appropriate personal protective equipment (PPE), testing patients for acute SARS-CoV-2 infection upon admission and rapid isolation of suspected COVID-19 cases in separate rooms. In the hospital complex surveyed here, the ER implemented these measures early on, possibly explaining the low seropositivity among these HCWs, despite the ER being a common entry point for symptomatic COVID-19 patients into hospitals [34].

The increased COVID-19 risk for HCWs working on ICUs, especially for nurses, indicates that patients with critical COVID-19 being treated on ICUs may pose a higher risk of contagion possibly due to individual patient contacts being more intense compared to other wards. Also, working as a nurse requires closer and longer patient contacts, which could serve as an explanation for the elevated COVID-19 risk ratio in this occupational group. In addition, specific characteristics in their work environment or socioeconomic factors may put nurses at higher risk.

HCWs reporting smoking behavior had a lower risk for seropositivity in multivariate analysis. A fraction of active smokers might have deliberately not reported their smoking behavior. This reporting bias could have lead to an underestimation of the protective effect of active smoking on the risk of SARS-CoV-2 infection in our analysis. Behavioral factors might explain the preventative effect of active smoking in HCWs, including the requirement to smoke outside the hospital that may have avoided high-risk exposures to colleagues in designated break areas and lunchrooms. However, direct antiviral effects related to smoking have also been reported [35, 36].

We showed that in resource-limited settings, a PCR-testing strategy for HCWs that focused on the presentation of symptoms and reporting of high-risk exposure, was sufficient to identify the majority of COVID-19 cases and prevent larger unrecognized outbreaks in the study population. However, if testing capacities are higher this strategy can be complemented by interval screening for acute SARS-CoV-2 infection, especially in the identified risk groups. Risk stratification in an unbiased decision tree, as shown in this study, may help refine screening efforts and enable more effective, personalized application of preventive measures.

This study was conducted directly after the first wave of the pandemic had subsided in the region. HCWs’ risk of SARS-CoV-2 infection was potentially increased during the early weeks of the pandemic due to limited PPE and PCR testing capacities, the need for rapid restructuring of units within the hospital and redeployment of HCWs to frontline positions [37]. Thus, risk factors reported here might not directly apply to later stages of the pandemic to the same extent. In turn, the COVID-19 seroprevalence at the start of the pandemic was generally low enabling a well-defined identification of hospital-specific rather than risk factors in the general population [26]. Participation rates were high among nurses (91.2%), and physicians (72.6%), but lower among other occupations such as cleaning personnel (18.3%) leading to risk assessments with limited confidence in the latter groups.

Of note, 19.2% (32/166) of seroconverted participants in our study reported having received only negative PCR results. We assume this represents the group of HCWs either returning from quarantine after COVID-19 or who had been tested PCR-negative during the incubation period [38]. The high specificities of the two anti-SARS-CoV-2 antibody detection assays used for screening (Elecsys^®^ 100%, and self-developed assay 99.9%) make false-positive antibody testing unlikely to explain this observation. Conversely, 21.8% (22/101) of participating HCWs did not seroconvert despite self-reporting a positive PCR test. Among others, this observation may be explained by reduced sensitivity of anti-SARS-CoV-2 antibody detection assays in asymptomatic and mild COVID-19 cases during the first weeks after infection.

54.8% of seropositive participants reported no high-risk contacts, suggesting that even professionals in the healthcare sector can be unaware of relevant exposures to SARS-CoV-2. Alternatively, deliberate underreporting of high-risk exposures may have occurred despite pseudonymized data collection. Moreover, HCWs returning from early COVID-19 hotspots in late February 2020 [39, 40], after the winter break in Southern Germany, may not have been aware of SARS-CoV-2 exposures during their vacation.

In summary, we identified several risk and protective factors for SARS-CoV-2 infection in HCWs related to high-risk exposures, profession, department, work unit, gender and behavior, as well as COVID-19-associated symptoms. Multivariate analysis underlined the importance of these factors, and risk stratification in an unbiased decision tree revealed subgroups within HCWs with distinct risk profiles. For the first time, we evaluated protective measures against SARS-CoV-2 spread and revealed that working from home was not effective, while a simple PCR-testing strategy was sufficient to detect the majority of COVID-19 cases among employees. Our findings suggest that future efforts to protect HCWs from COVID-19, including, training programs, screening for acute infection, quarantining, and vaccination, should be risk factor-driven.

## Supplementary Information

Below is the link to the electronic supplementary material.

Supplementary file1 (PDF 1259 KB)

## Data Availability

The datasets used and/or analyzed during the current study are available from the corresponding author on reasonable request.
