# Supplementary material for: In-depth profiling of COVID-19 risk factors and preventive measures in healthcare workers
Source: Infection. 2021 Aug 11;50(2):381–94. doi: 10.1007/s15010-021-01672-z (PMC8354838; doi:10.1007/s15010-021-01672-z)
Supplement: Supplementary file 1 — Supplementary file1 (PDF 1259 KB) [file 15010_2021_1672_MOESM1_ESM.pdf]

Supplementary Information:

In-depth profiling of COVID-19 risk factors and preventive measures in healthcare workers

Paul R Wratil, Niklas A Schmacke, Andreas Osterman, Tobias Weinberger, Jochen Rech, Burak Karakoc, Mira Zeilberger, Julius Steffen, Tonina T Mueller, Patricia M Spaeth, Marcel Stern, Manuel Albanese, Hella Thun, Julia Reinbold, Benedikt Sandmeyer, Philipp Kressirer, Béatrice Grabein, Peter Falkai, Kristina Adorjan, Veit Hornung, Lars Kaderali, Matthias Klein, Oliver T Keppler

Corresponding Authors:

Paul R. Wratil (wratil@mvp.lmu.de)

Oliver T. Keppler (keppler@mvp.lmu.de)

Supplementary figure legends

Supplementary Fig. 1. Testing and calling strategy used to determine anti-SARS-CoV-2 antibody status. (a) All samples were screened with both a commercially available and a self-developed SARS-CoV-2 antibody test. Three samples were determined positive with only positive Elecsys results. Five indeterminate samples were unavailable for additional testing and were called negative. (b) Agreement of the Elecsys and self-developed IgG assay across all study samples. Two samples were positive in the self-developed IgG assay but called negative by the Elecsys test (red).

Supplementary Fig. 2. Neutralizing activity in SARS-CoV-2 PCR$^+$ or Ab$^+$ sera. (a) Overview of neutralization assay procedure. (b) Number of PCR$^+$ or Ab$^+$ HCWs by their SARS-CoV-2 neutralizing activity categorized as follows: "none": $IC_{50} < 10$; "weak": $IC_{50} < 90$; "medium": $IC_{50} < 270$; "strong": $IC_{50} < 2430$; "very strong": $IC_{50} > 2430$. (c) SARS-CoV-2 neutralizing activity of serum from PCR$^+$ or Ab$^+$ participants by how long ago they were first PCR-tested. Black triangles represent the strength of neutralizing activity from "none" to "very strong" as in (b). (d) SARS-CoV-2 neutralizing ability by antibody titer in serum as measured by the Elecsys assay. *P*-values throughout the figure

were calculated using Kendall's τ statistic.

Supplementary Fig. 3. High-risk exposures in HCWs including multiple exposure types. (a) Related to Fig. 2a, risk of SARS-CoV-2 seroconversion of staff members by self-reported instances of different types of high-risk exposure. Multiple answers are included in each respective group. Lines indicate 95% CIs.

Supplementary Fig. 4. Work environments and associated risks in study participants. (a) Numbers of nurses, physicians and other patient-facing HCWs who reported patient contacts on the indicated clinical units compared to how many patient contacts per day they reported on average. Multiple mentions for units were possible. (b) Percent seropositivity of nurses (blue bars), physicians (orange bars) and others with patient contact (red bars) by units on which they reported patient contacts. Multiple mentions for units were possible. Lines indicate 95% CIs. The dashed line indicates the overall seropositivity in the study population (2.2%)

Supplementary Fig. 5. High-risk exposures in the hospital are more frequent on COVID-19 units, seropositivity rates among participants from different study centres and among those with medical preconditions. (a) Relative proportion of high-risk exposures in indicated settings among study participants by their anti-SARS-CoV-2 antibody status and whether they reported to have worked on COVID-19 units. HCWs reporting high-risk exposures in multiple settings are shown as "mixed". (b) Percent anti-SARS-CoV-2 Ab^+ HCWs reporting working primarily at one of the two study centres relative to all HCWs primarily working at that centre divided by being deployed to COVID-19 units or not. (c) Percent anti-SARS-CoV-2 Ab^+ HCWs reporting any of the indicated medical conditions relative to all HCWs who reported the given condition. Study participants reporting multiple conditions are included under each condition. Lines in (b) and (c) indicate 95% CIs.

Supplementary Fig. 6. Symptom combinations among study participants. (a) Absolute frequency of reported symptom combinations among study participants by anti-SARS-CoV-2 antibody status. Colors indicate how many symptoms were in a given combination. The most specific symptom combination was taste disorder, fever, headache and fatigue.

Supplementary Fig. 7. Evaluation of working from home as a precaution. (a) Percentage of staff members reporting high-risk exposures in their community by whether they were working from home as a precaution. (b) Percent staff members from administrative and research occupations by whether they worked from home as a precaution and whether they self-reported high-risk exposures of the indicated type. Focussing on this subgroup, for which working from home was generally available, allowed us to directly compare HCWs in the same occupation who worked from home as a precaution with those who did not. *P*-values were calculated using Fisher's exact test. (c) Percentage of high-risk exposures in the hospital reported to the occupational health office by occupation of the reporting HCW.

Supplementary Fig. 8. Symptoms and high-risk exposures as indications for PCR testing in health care workers. (a) Likelihood of study participants reporting no high-risk exposure (2,482) to get PCR-tested based on which symptoms they indicated. Numbers in braces represent staff members from this group who did get PCR-tested and reported the respective symptom.

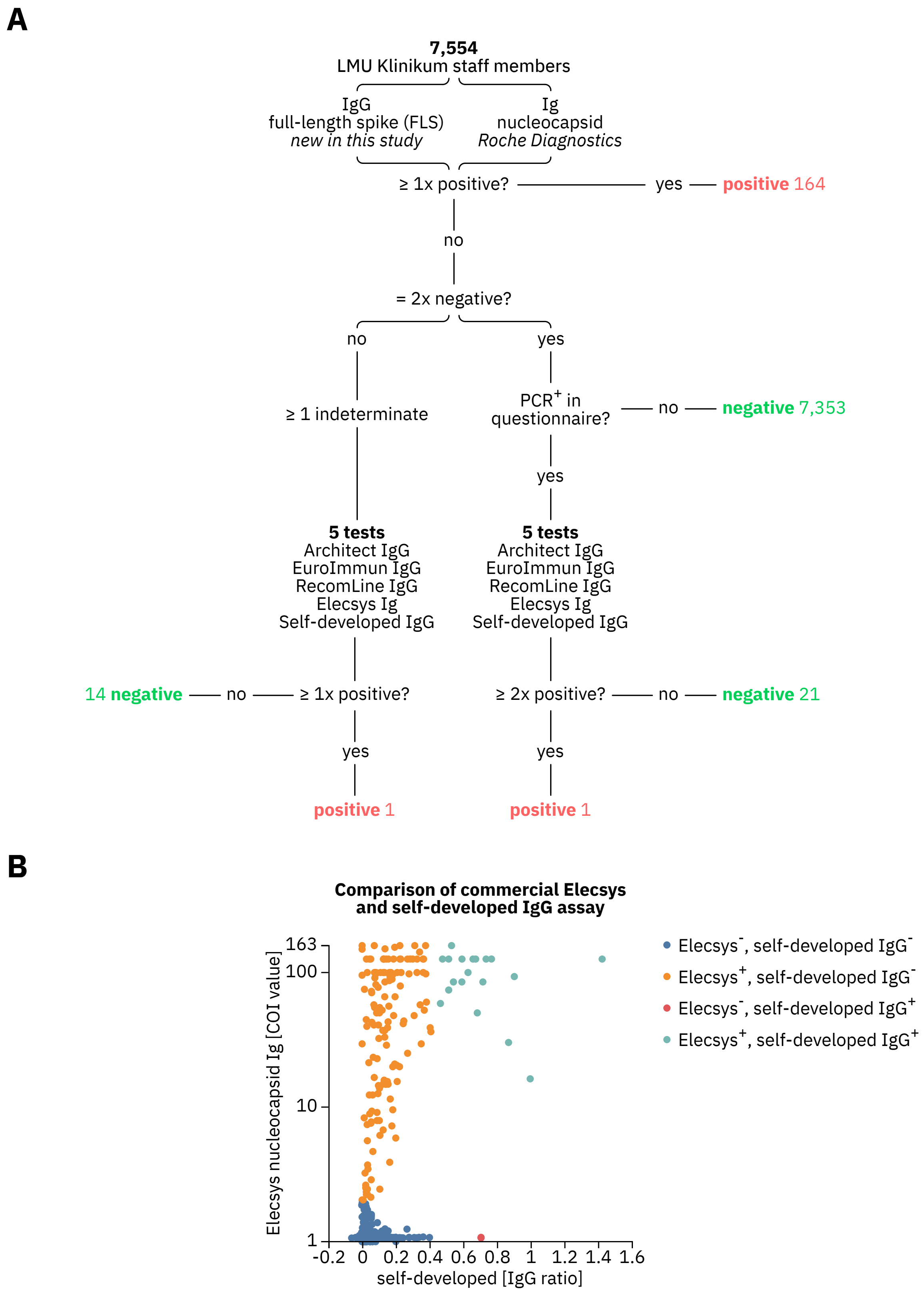

Supplementary Figure 1

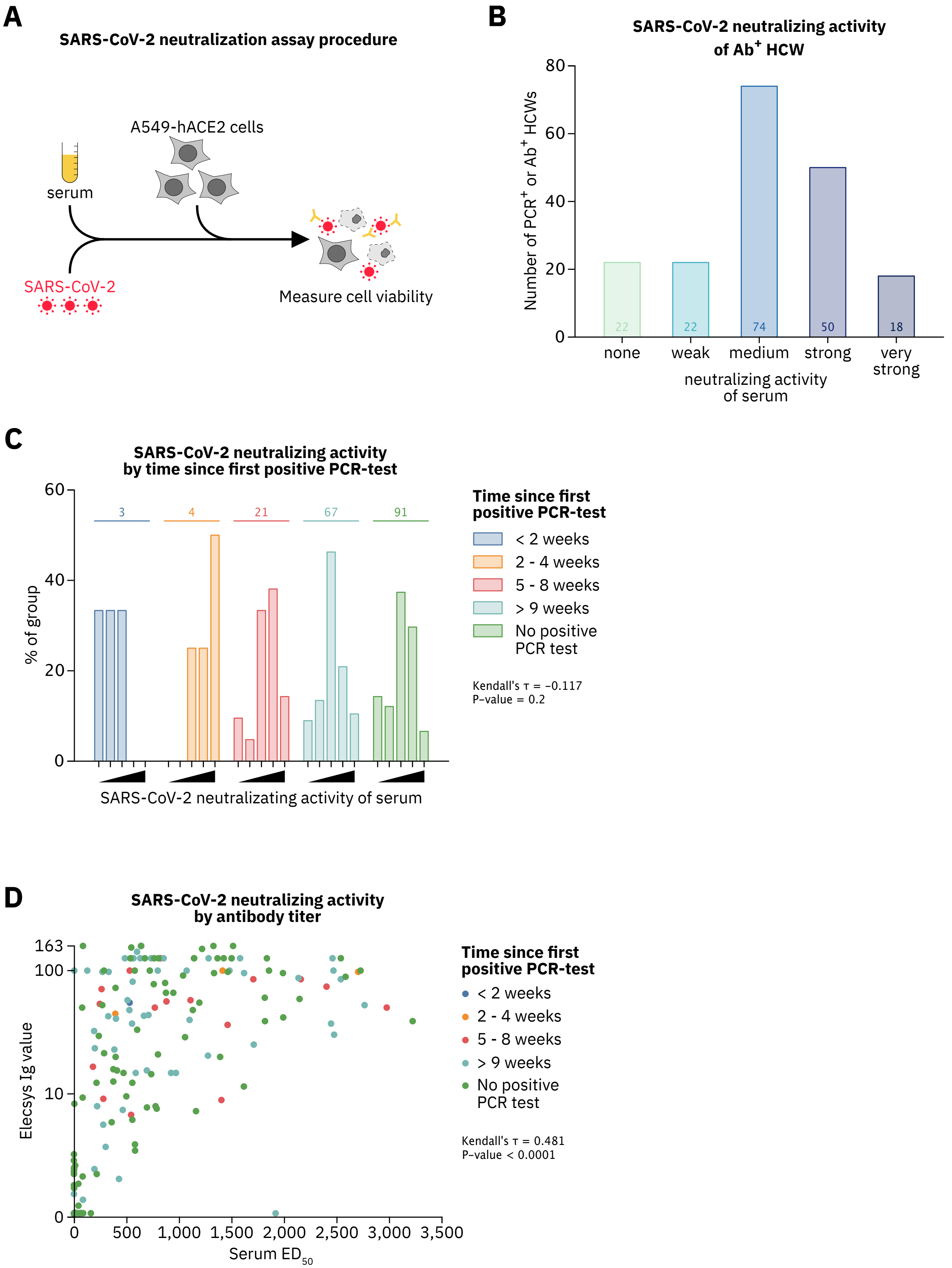

Supplementary Figure 2

A

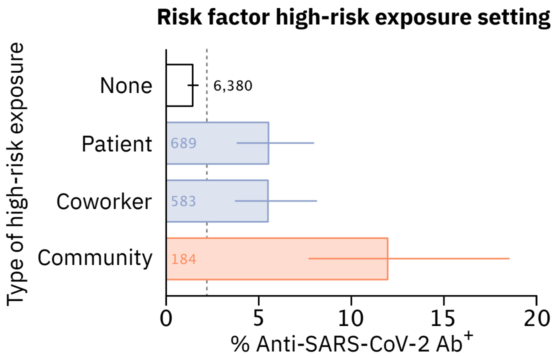

Supplementary Figure 3

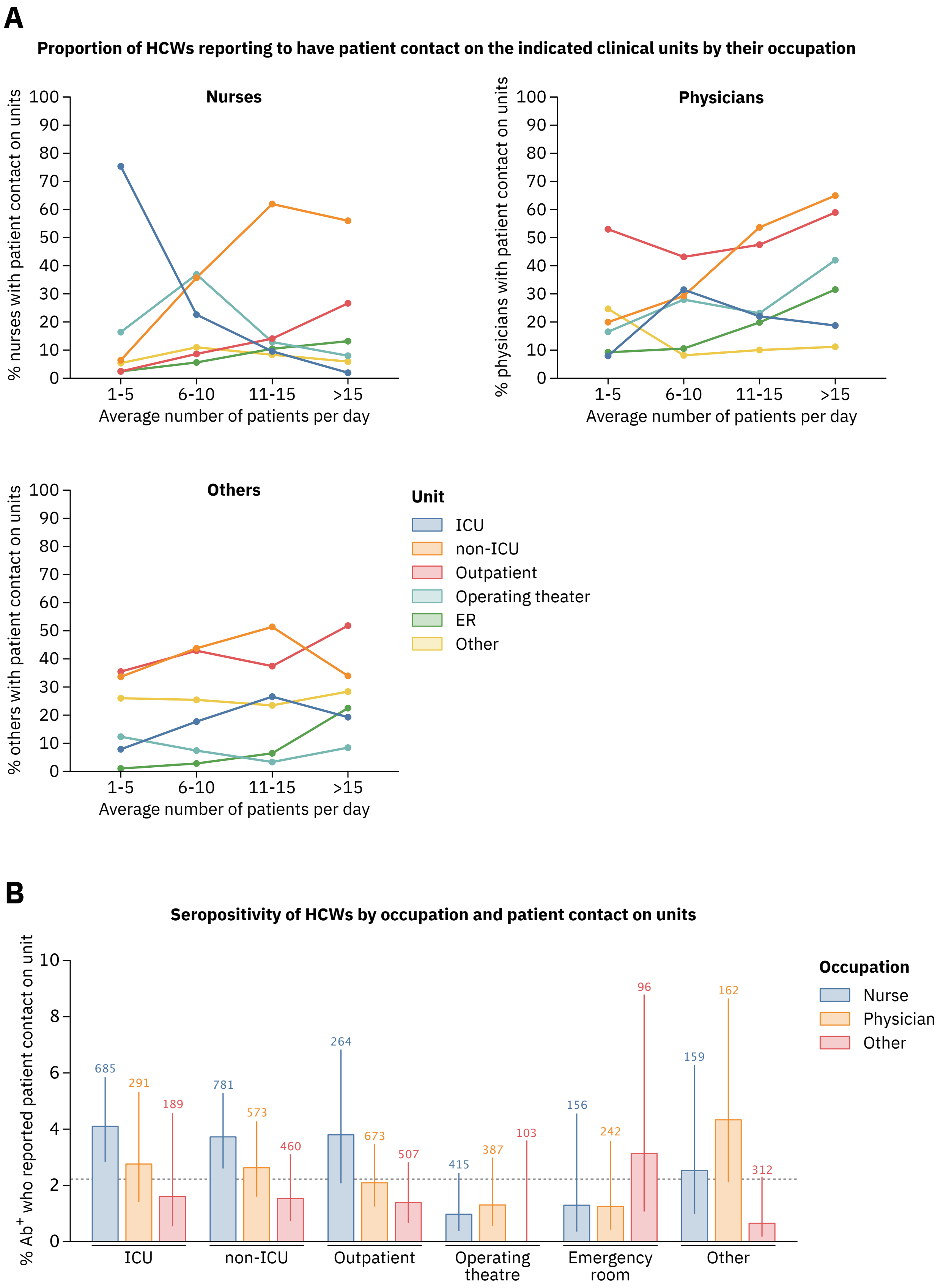

Supplementary Figure 4

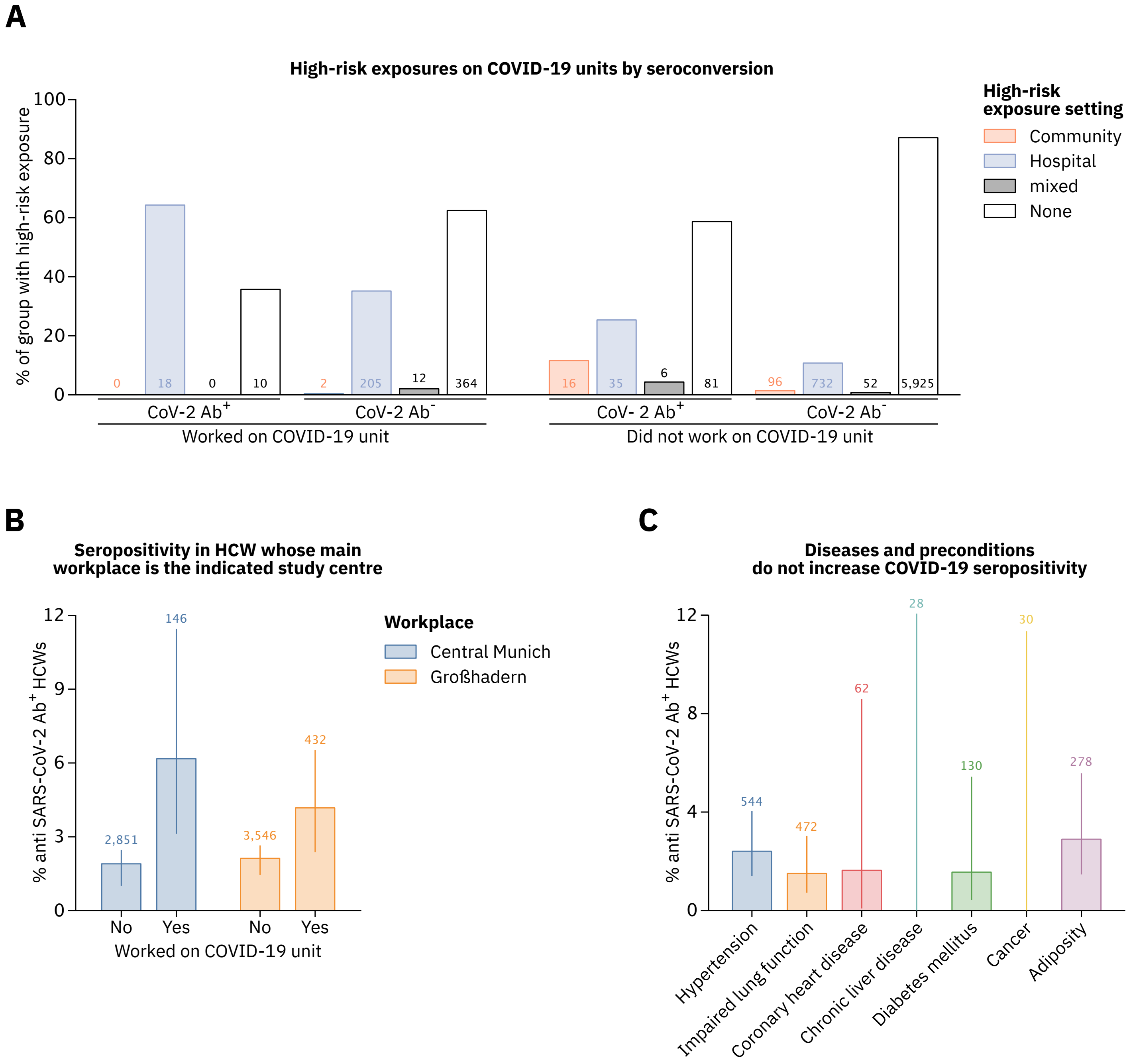

Supplementary Figure 5

A

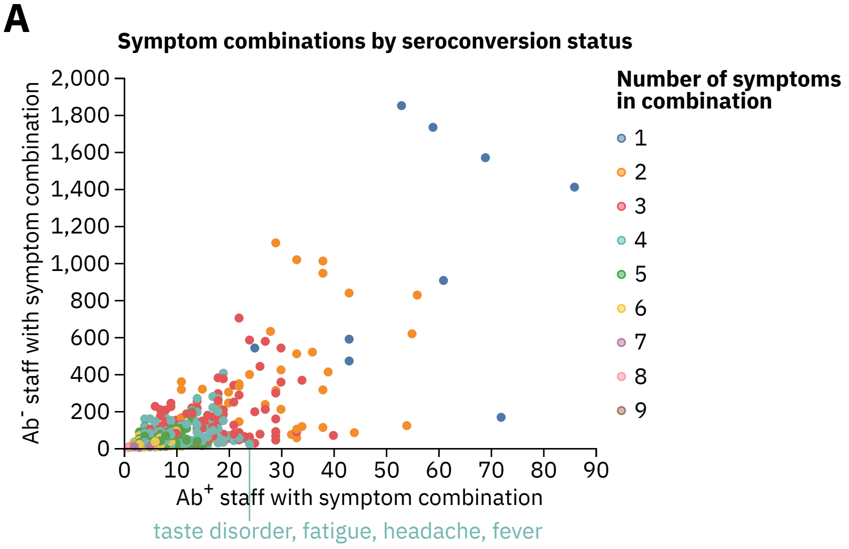

Supplementary Figure 6

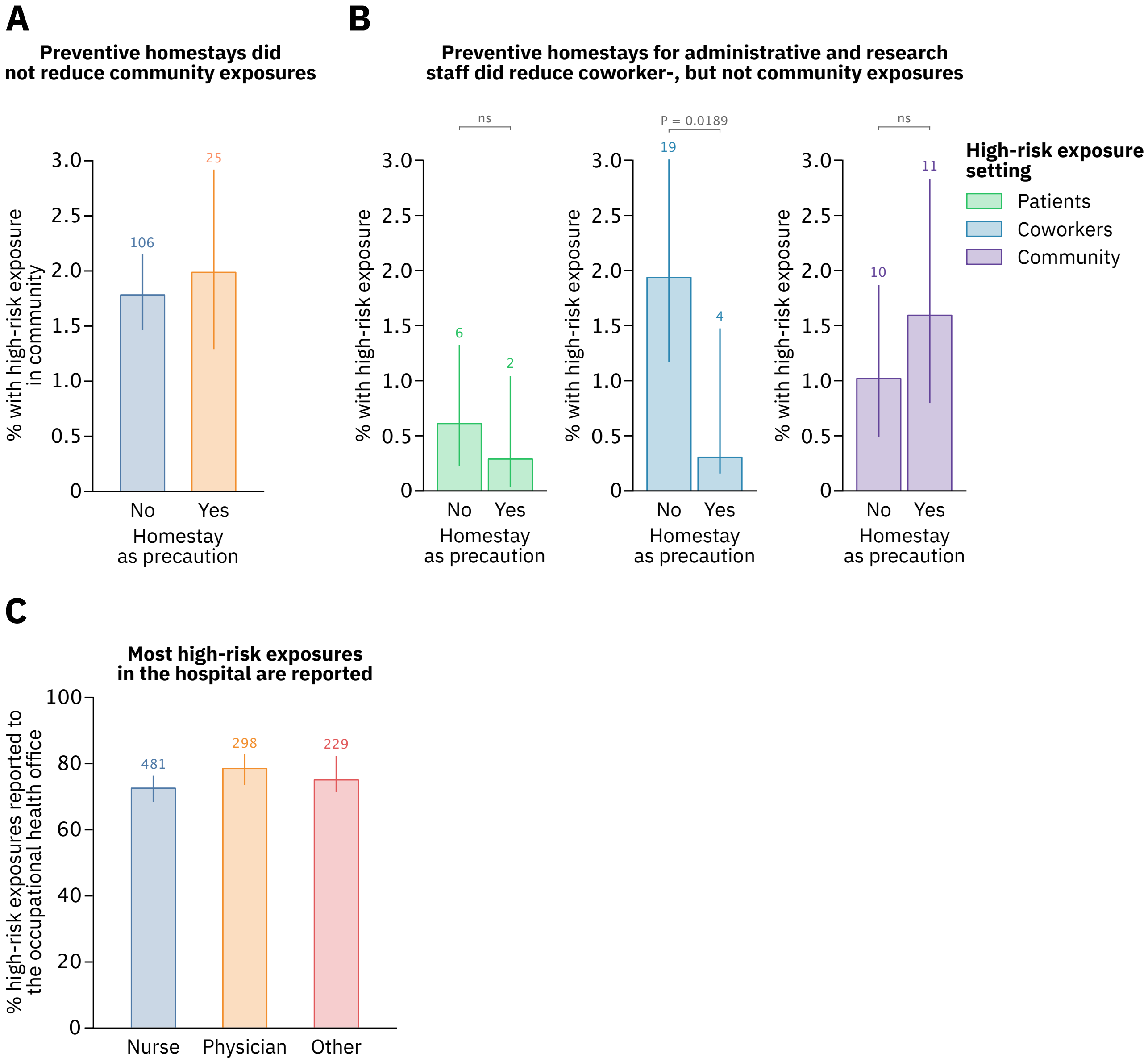

Supplementary Figure 7

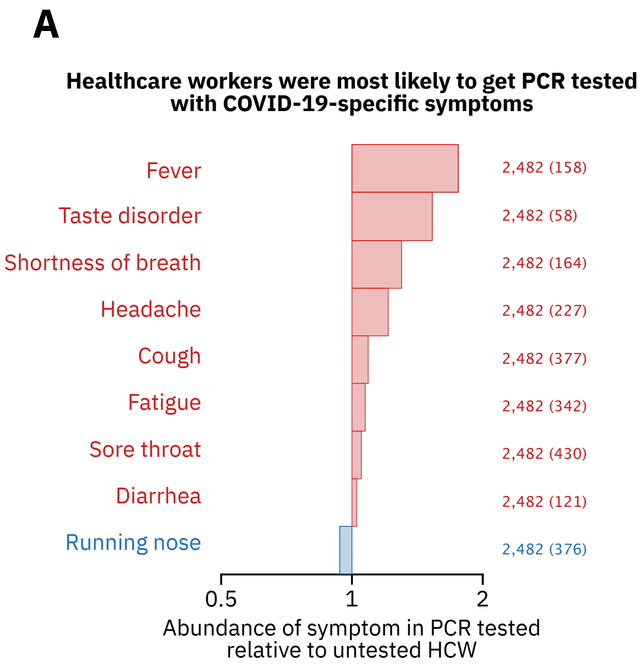

Supplementary Figure 8

Supplementary Table 1 (part 1). Study questionnaire compared to anti-SARS-CoV-2 antibody status of 7,554 health care workers participating in the study.

Questions	Answers	Anti-SARS-CoV-2 Ab	
		Pos. / total	%
Please state your age.	≤ 30 Y	64 / 2,170	2.95
	31 - 40 Y	39 / 1,951	2.00
	41 - 50 Y	29 / 1,430	2.03
	51 - 60 Y	23 / 1,467	1.57
	> 60 Y	11 / 536	2.05
Please state your gender.	male	51 / 2,118	2.41
	female	115 / 5,431	2.12
	third gender	0 / 5	0.00
Do you take immunosuppressants?	yes	4 / 177	2.26
	no	162 / 7,377	2.20
Were you vaccinated against Influenza in flu season 2019/20?	yes	67 / 2,630	2.55
	no	99 / 4,924	2.01
Are you planning to get vaccinated against Influenza in flu season 2020/21?	yes	85 / 3,390	2.51
	no	49 / 2,719	1.80
	undecided	32 / 1,445	2.21
Would you get vaccinated against SARS-CoV-2 if there was an efficient vaccination available with few side effects?	yes	85 / 4,397	1.93
	no	26 / 913	2.85
	undecided	55 / 2,240	2.46
Do you have any relevant pre-existing medical conditions?	yes	20 / 1,117	1.79
	no	146 / 6,437	2.27
For participants with relevant pre-existing medical conditions: I have the following pre-existing medical conditions:	hypertension	13 / 544	2.39
	lung disease (e.g. asthma, COPD, chronic bronchitis)	7 / 472	1.48
	coronary heart disease	1 / 62	1.61
	chronic liver disease	0 / 28	0.00
	diabetes mellitus	2 /130	1.54
	active cancer	0 / 30	0.00
	obesity	8 / 278	2.88
Do you smoke?	yes	16 / 1,423	1.12
	no	132 / 5,482	2.41
	I stopped smoking within the last 10 Y	18 / 649	2.77
Including yourself, how many adults live in your household?	1	56 / 2,355	2.38
	2	75 / 3,699	2.03
	> 2	35 / 1,500	2.33
How many children under the age of 18 Y live your household?	1	16 / 1,007	1.59
	2	14 / 828	1.69
	3	5 / 176	2.84
	4	1 / 38	2.63
	none	130 / 5,505	2.36
Have you been tested against SARS-CoV-2 using virus specific PCR?	positive (at least once, if multiple tests)	79 / 101	78.22
	negative (all tests, if multiple tests)	16 / 1,423	1.12
	not tested	55 / 5,610	0.98
For positive tested participants: When have you been tested positive for the first time?	< 2 weeks ago	1 / 3	33.33
	2 - 4 weeks ago	4 / 4	100.00
	5 - 8 weeks ago	19 / 22	86.36
	> 9 weeks ago	55 / 72	76.39
For positive tested participants: Did you show any symptoms during that infection?	yes	71 / 84	84.52
	no	8 / 18	44.44

Supplementary Table 1 (part 2). Study questionnaire compared to anti-SARS-CoV-2 antibody status of 7,554 health care workers participating in the study.

Questions	Answers	Anti-SARS-CoV-2 Ab	
		**Pos. / total**	**%**
In which department/institute-type do you work?	conservative	78 / 2,164	3.60
	surgical	31 / 1,975	1.57
	pediatrics	16 / 908	1.76
	other clinical	45 / 1,488	3.02
	non-clinical	6 / 1019	0.59
In the last weeks, in which center did you work primarily?	Großhadern	93 / 3,978	2.34
	downtown	63 / 2,997	2.10
	both	3 / 251	1.20
	none of the above	7 / 328	2.13
Are you directly involved in patient care?	yes	123 / 4,729	2.60
	no	34 / 2,825	1.20
For participants working in patient care: I have the following profession:	nurse	68 / 2,185	3.11
	medical technician	3 / 303	0.99
	physical therapist/psychotherapist/occu-pational therapist/speech therapist	5 / 272	1.83
	physician	38 / 1,345	2.83
	other profession	9 / 624	1.44
For participants working in patient care: How many patients do you see per day on average?	< 5 patients	33 / 1,293	2.55
	5 - 10 patients	27 / 1,397	1.93
	11 - 15 patients	21 / 736	2.85
	> 15 patients	42 / 1,303	3.22
For participants working in patient care: Where do you primarily have direct contact with patients?	outpatient unit	31 / 1,444	2.15
	emergency unit	8 / 494	1.62
	normal care unit	51 / 1,814	2.81
	ICU/monitoring unit	39 / 1,165	3.35
	operation theater	9 / 905	0.99
	other	13 / 633	2.05
For participants working in patient care: Did you work on a COVID-19 ward?	yes	28 / 611	4.58
	no	95 / 4,118	2.31
For participants not working in patient care: What is your working area:	transportation	1 / 28	3.57
	cleaning personnel	4 / 119	3.36
	office work/ IT	15 / 822	1.82
	research	12 / 977	1.23
	medical institute without direct patient contact	2 / 210	0.95
	other	9 / 669	1.35
For participants not working in patient care: I had the following contacts with patients/patient material:	short patient contacts (e.g. transportation, cleaning, in the office)	8 / 634	1.26
	contact with patient material (e.g. in the laboratory)	1 / 360	0.28
	no relevant contact to patients/ patient material	34 / 1,831	1.86
In the last three months, have you worked from home for at least 1 week?	yes, as a prophylactic measure	22 / 1,377	1.60
	yes, because I was in quarantine (infected with SARS-CoV-2 or contact to COVID-19 patient)	37 / 164	22.56
	no	107 / 6,013	1.78

Supplementary Table 1 (part 3). Study questionnaire compared to anti-SARS-CoV-2 antibody status of 7,554 health care workers participating in the study.

Questions	Answers	Anti-SARS-CoV-2 Ab	
		**Pos. / total**	**%**
How long did you work from home, or how long were you quarantined?	< 1 week	5 / 362	1.38
	1 week	1 / 212	0.47
	2 weeks	31 / 364	8.51
	3 weeks	12 / 158	7.59
	4 weeks	8 / 176	4.55
	5 weeks	5 / 77	6.49
	> 5 weeks	16 / 509	3.14
	no answer	88 / 5,696	1.54
Do you suspect that you have been exposed to SARS-CoV-2 in any of the following scenarios?	patient contact	97 / 3,798	2.55
	contact to infected colleagues	87 / 4,141	2.10
	private contact (not at work)	83 / 3,966	2.09
	no increased exposure	8 / 1,382	0.58
Did you have contact to SARS-CoV-2 infected patients and at least one of the following criteria was met?<br>- at least 15 min face-to-face contact without protective gear (at least protective mask worn by patient and study participant)<br>- direkt contact to body fluids, especially fluids originating from the respiratory tract<br>- performing aerosol forming measures (e.g. tracheal aspiration)<br>- medical examination or nursing without protective gear and < 2 m distance to patient	yes	38 / 689	5.52
	no	128 / 6,865	1.88
If a contact to a SARS-CoV-2 infected patient following the criteria above occured: Was this contact reported to the occupational health office?	yes	33 / 461	7.16
	no	5 / 228	2.19
Did you have contact to SARS-CoV-2 infected colleagues and ≥ 1 of the following criteria was met?<br>- at least 15 min face-to-face contact without protective gear (at least protective mask worn by patient and study participant)<br>- direkt contact to body fluids, especially fluids originating from the respiratory tract	yes	32 / 583	5.49
	no	134 / 6,971	1.92
If a contact to a SARS-CoV-2 infected colleague following the criteria above occured: Was this contact reported to the occupational health office?	yes	31 / 503	6.16
	no	1 / 80	1.25
Did you have contact to SARS-CoV-2 infected individuals outside of work and ≥ 1 of the following criteria was met?<br>- at least 15 min face-to-face contact without protective gear (at least protective mask worn by patient and study participant)<br>- direkt contact to body fluids, especially fluids originating from the respiratory tract	yes	22 / 184	11.96
	no	144 / 7,370	1.95

Supplementary Table 1 (part 4). Study questionnaire compared to anti-SARS-CoV-2 antibody status of 7,554 health care workers participating in the study.

Questions	Answers	Anti-SARS-CoV-2 Ab	
		**Pos. / total**	**%**
If a contact to a SARS-CoV-2 infected individual outside of work following the criteria above occured: Were you contacted by the public health authority?	yes	18 / 80	22.50
	no	4 / 104	3.85
In the last three months, did you experience any cold-like symptoms?	yes	120 / 2,986	4.02
	no	46 / 4,568	1.01
If cold-like symptoms were experienced: Which of the following symptoms did you experience?	fever > 38 °C	43 / 517	8.32
	cough	69 / 1,641	4.20
	shortness of breath	43 / 635	6.77
	fatigue	86 / 1499	5.74
	running nose	59 / 1,795	3.29
	sore throat	53 / 1,906	2.78
	unusual headache	61 / 970	6.29
	diarrhea	25 / 569	4.39
	taste disorder	72 / 242	29.75

Supplementary Table 2 (part 1). Study questionnaire compared to anti-SARS-CoV-2 antibody status of 7,554 health care workers participating in the study (German).

Questions	Answers	Anti-SARS-CoV-2 Ab	
		Pos. / total	%
Bitte geben Sie hier Ihr Alter an.	≤ 30 Jahre	64 / 2,170	2.95
	31 - 40 Jahre	39 / 1,951	2.00
	41 - 50 Jahre	29 / 1,430	2.03
	51 - 60 Jahre	23 / 1,467	1.57
	> 60 Jahre	11 / 536	2.05
Bitte geben Sie hier Ihr Geschlecht an.	männlich	51 / 2,118	2.41
	weiblich	115 / 5,431	2.12
	divers	0 / 5	0.00
Nehmen Sie immunsuppressive oder immunmodulierende Medikamente?	ja	4 / 177	2.26
	nein	162 / 7,377	2.20
Haben Sie sich in der Saison 2019/2020 gegen Influenza impfen lassen?	ja	67 / 2,630	2.55
	nein	99 / 4,924	2.01
Planen Sie, sich in der nächsten Saison 2020/2021 gegen Influenza impfen zu lassen?	ja	85 / 3,390	2.51
	nein	49 / 2,719	1.80
	vielleicht	32 / 1,445	2.21
Bei Verfügbarkeit eines nebenwirkungsarmen und effizienten Impfstoffes gegen SARS-CoV-2 würde ich mich impfen lassen?	ja	85 / 4,397	1.93
	nein	26 / 913	2.85
	vielleicht	55 / 2,240	2.46
Liegen bei Ihnen relevante Vorerkrankungen vor?	ja	20 / 1,117	1.79
	nein	146 / 6,437	2.27
Bei Probanden mit relevanten Vorerkrankungen: Bei mir liegen folgende Vorerkrankungen vor:	Bluthochdruck	13 / 544	2.39
	Lungenerkrankungen (z.B. Asthma, COPD, chronische Bronchitis)	7 / 472	1.48
	Koronare Herzerkrankung	1 / 62	1.61
	Chronische Lebererkrankung	0 / 28	0.00
	Diabetes mellitus	2 /130	1.54
	Aktive Krebserkrankung	0 / 30	0.00
	Adipositas	8 / 278	2.88
Planen Sie, sich in der nächsten Saison 2020/2021 gegen Influenza impfen zu lassen?	ja	16 / 1,423	1.12
	nein	132 / 5,482	2.41
	Ex-Raucher (in den letzten 10 Jahren)	18 / 649	2.77
Wie viele erwachsene Personen leben insgesamt in Ihrem Haushalt (mit Ihnen eingeschlossen)?	1	56 / 2,355	2.38
	2	75 / 3,699	2.03
	> 2	35 / 1,500	2.33
Wie viele Kinder unter 18 Jahre leben in Ihrem Haushalt?	1	16 / 1,007	1.59
	2	14 / 828	1.69
	3	5 / 176	2.84
	4	1 / 38	2.63
	keine	130 / 5,505	2.36
Wurden Sie bereits per PCR (Abstrich) auf SARS-CoV-2 getestet?	positiv (mindestens einmal, falls Mehrfachtestung)	79 / 101	78.22
	negativ (immer negativ, falls Mehrfachtestung)	16 / 1,423	1.12
	nicht getestet	55 / 5,610	0.98
Für positiv getestete Teilnehmer: Wann wurden Sie zum 1. Mal positiv getestet?	vor < 2 Wochen	1 / 3	33.33
	vor 2 - 4 Wochen	4 / 4	100.00
	vor 5 - 8 Wochen	19 / 22	86.36
	vor > 9 Wochen	55 / 72	76.39

Supplementary Table 2 (part 2). Study questionnaire compared to anti-SARS-CoV-2 antibody status of 7,554 health care workers participating in the study (German).

Questions	Answers	Anti-SARS-CoV-2 Ab	
		Pos. / total	%
Für positiv getestete Teilnehmer: Hatten sie bei dieser Infektion Symptome?	ja	71 / 84	84.52
	nein	8 / 18	44.44
In welcher Klinik/Organisationseinheit sind Sie am Klinikum beschäftigt?	konservativ	78 / 2,164	3.60
	chirurgisch	31 / 1,975	1.57
	Pädiatrie	16 / 908	1.76
	andere klinische Bereiche	45 / 1,488	3.02
	nicht-klinische Bereiche	6 / 1019	0.59
Waren Sie in den letzten Wochen primär in der Innenstadt oder in Großhadern tätig?	Großhadern	93 / 3,978	2.34
	Innenstadt	63 / 2,997	2.10
	sowohl als auch	3 / 251	1.20
	weder noch	7 / 328	2.13
Arbeiten Sie in der unmittelbaren Patientenversorgung?	ja	123 / 4,729	2.60
	nein	34 / 2,825	1.20
Für Teilnehmer aus der direkten Patientversogung: In welcher?	Pflege	68 / 2,185	3.11
	Technischer Assistenzberuf (MTA/MTRA/…)	3 / 303	0.99
	Physiotherapie/Psychotherapie/Ergotherapie/Logopädie	5 / 272	1.83
	Arzt/Ärztin	38 / 1,345	2.83
	andere	9 / 624	1.44
Für Teilnehmer aus der direkten Patientversogung: Wieviele Patienten behandeln Sie durchschnittlich pro Tag?	< 5 Patienten	33 / 1,293	2.55
	5 - 10 Patienten	27 / 1,397	1.93
	11 - 15 Patienten	21 / 736	2.85
	> 15 Patienten	42 / 1,303	3.22
Für Teilnehmer aus der direkten Patientversogung: Wo sehen Sie überwiegend Patienten?	Ambulanz	31 / 1,444	2.15
	Notaufnahme	8 / 494	1.62
	Normalstation	51 / 1,814	2.81
	Intensivstation/IMC	39 / 1,165	3.35
	OP	9 / 905	0.99
	andere	13 / 633	2.05
Für Teilnehmer aus der direkten Patientversogung: Waren Sie auf einer COVID-Schwerpunktstation eingesetzt?	ja	28 / 611	4.58
	nein	95 / 4,118	2.31
Für Teilnehmer außerhalb der direktion Patientenversorgung: In welchem Bereich sind Sie tätig?	Transport	1 / 28	3.57
	Reinigung	4 / 119	3.36
	Büro/EDV	15 / 822	1.82
	Forschung	12 / 977	1.23
	Medizinisches Institut ohne direkten Patientenkontakt	2 / 210	0.95
	anderer	9 / 669	1.35
Für Teilnehmer außerhalb der direktion Patientenversorgung: Ich bin wie folgt mit Patienten/Material in Kontakt gekommen	Kurze Kontakte mit Patienten (z.B. Transport, Reinigung, Sekretariat)	8 / 634	1.26
	Kontakt mit Patientenmaterial (z.B. im Labor)	1 / 360	0.28
	Kein relevanter Kontakt zu Patienten/Patientenmaterial	34 / 1,831	1.86
Waren Sie in den letzten 3 Monaten mindestens 1 Woche durchgehend im Home Office tätig?	ja, prophylaktisch	22 / 1,377	1.60
	ja, da in Quarantäne (Kontakt zu COVID Patient oder selbst infiziert)	37 / 164	22.56
	nein	107 / 6,013	1.78

Supplementary Table 2 (part 3). Study questionnaire compared to anti-SARS-CoV-2 antibody status of 7,554 health care workers participating in the study (German).

Questions	Answers	Anti-SARS-CoV-2 Ab	
		Pos. / total	%
Wie lange war die Dauer des Home Office oder der Quarantäne?	< 1 Woche	5 / 362	1.38
	1 Woche	1 / 212	0.47
	2 Wochen	31 / 364	8.51
	3 Wochen	12 / 158	7.59
	4 Wochen	8 / 176	4.55
	5 Wochen	5 / 77	6.49
	> 5 Wochen	16 / 509	3.14
	keine Antwort	88 / 5,696	1.54
In welcher Situationen können Sie sich vorstellen, Kontakt mit dem Virus gehabt zu haben?	Patientenkontakt	97 / 3,798	2.55
	Kontakt mit Mitarbeitern	87 / 4,141	2.10
	Kontakt außerhalb der Arbeit	83 / 3,966	2.09
	keine erhöhte Exposition	8 / 1,382	0.58
Hatten Sie wissentlich Kontakt zu Patienten, die positiv für SARS-CoV-2 getestet wurden? Mindestens eines der folgenden Kriterien muss erfüllt sein<br><br>- mindestens 15-minütiger Gesichts- ("face-to-face") Kontakt ohne Schutzausrüstung (mindestens MNS bei Patient und Mitarbeiter), z.B. im Rahmen eines Gesprächs<br><br>- direkter Kontakt zu Sekreten oder Körperflüssigkeiten, insbesondere zu respiratorischen Sekreten, wie z. B. Kontakt zu Erbrochenem, Mund-zu-Mund Beatmung, Anhusten, Anniesen etc.<br><br>- Durchführung aerosolbildender Maßnahmen (z.B. Absaugen)<br><br>- Kontakt zum bestätigten COVID-19-Fall im Rahmen von Pflege oder medizinischer Untersuchung (< 2m), ohne verwendete Schutzausrüstung.	ja	38 / 689	5.52
	nein	128 / 6,865	1.88
Falls ein Kontakt zu einem mit SARS-CoV-2 infizierten Patienten auftrat, der die in der Vorfrage genannten Kriterien erfüllt: Wurde der Kontakt dem betriebsärztlichen Dienst gemeldet?	ja	33 / 461	7.16
	nein	5 / 228	2.19
Hatten Sie wissentlich Kontakt zu auf SARS-CoV-2 positiv getesteten Mitarbeitern? Mindestens eines der folgenden Kriterien muss erfüllt sein:<br><br>- mindestens 15-minütiger Gesichts- ("face-to-face") Kontakt ohne Schutzausrüstung (mindestens MNS bei Patient und Mitarbeiter), z.B. im Rahmen eines Gesprächs<br><br>- direktem Kontakt zu Sekreten oder Körperflüssigkeiten, insbesondere zu respiratorischen Sekreten, wie z. B. Küssen, Anhusten, Anniesen, etc.	ja	32 / 583	5.49
	nein	134 / 6,971	1.92

Supplementary Table 2 (part 4). Study questionnaire compared to anti-SARS-CoV-2 antibody status of 7,554 health care workers participating in the study (German).

Questions	Answers	Anti-SARS-CoV-2 Ab	
		**Pos. / total**	**%**
Falls ein Kontakt zu einem mit SARS-CoV-2 infizierten Kollegen auftrat, der die in der Vorfrage genannten Kriterien erfüllt: Wurde der Kontakt dem betriebsärztlichen Dienst gemeldet?	ja	31 / 503	6.16
	nein	1 / 80	1.25
Hatten Sie wissentlich Kontakt zu auf SARS-CoV-2 positiv getesteten Personen im privaten Umfeld? Mindestens eines der folgenden Kriterien muss erfüllt sein:<br><br>- mindestens 15-minütigem Gesichts- ("face-to-face") Kontakt ohne Schutzausrüstung (mindestens MNS bei beiden Personen), z.B. im Rahmen eines Gesprächs<br><br>- direktem Kontakt zu Sekreten oder Körperflüssigkeiten, insbesondere zu respiratorischen Sekreten, wie z. B. Küssen, Kontakt zu Erbrochenem, Mund-zu-Mund Beatmung, Anhusten, Anniesen, etc.	ja	22 / 184	11.96
	nein	144 / 7,370	1.95
Falls ein Kontakt zu einer mit SARS-CoV-2 infizierten Person im privaten Umfeld auftrat, der die in der Vorfrage genannten Kriterien erfüllt: Wurden Sie in der Folge vom Gesundheitsamt kontaktiert?	ja	18 / 80	22.50
	nein	4 / 104	3.85
Hatten Sie in den letzten 3 Monaten erkältungsähnliche Symptome?	ja	120 / 2,986	4.02
	nein	46 / 4,568	1.01
Falls erkältungsähnliche Symptome auftraten: Welche der folgenden Symptome sind aufgetreten?	Fieber > 38 °C	43 / 517	8.32
	Husten	69 / 1,641	4.20
	Kurzatmigkeit	43 / 635	6.77
	verstärkte Müdigkeit	86 / 1499	5.74
	Schnupfen	59 / 1,795	3.29
	Halsschmerzen	53 / 1,906	2.78
	Kopfschmerzen (die so nicht für sie üblich sind)	61 / 970	6.29
	Durchfall	25 / 569	4.39
	Geschmacksstörungen	72 / 242	29.75

Supplementary Table 3. Assay specificity determination of different anti-SARS-CoV-2 antibody detection assays in serum samples from healthy adult blood donors and children/adolescents (< 18 years) collected prior to december 2019.

Assay	Sample description	False positve / Total	Specificity (%)	95% CI (%)
Architect Assay	Adults	4 / 888	99.55	98.85 - 99.82
	Children	0 / 264	100.00	98.57 - 100.00
	**Total**	**4 / 1,152**	**99.65**	**99.11 - 99.86**
EuroImmun Assay	Adults	15 / 888	98.31	97.23 - 98.97
	Children	7 / 264	97.35	94.63 - 98.71
	**Total**	**22 / 1,152**	**98.09**	**97.13 - 98.74**
Self-Developed Assay	Adults	1 / 888	99.89	99.36 - 99.99
	Children	0 / 264	100.00	98.57 - 100.00
	**Total**	**1 / 1,152**	**99.91**	**99.51 - 100.00**
recomLine Assay	Adults	3 / 184	98.37	95.32 - 99.44
	Children	2 / 153	98.69	95.36 - 99.64
	**Total**	**5 / 337**	**98.52**	**96.57 - 99.36**
Elecsys Assay	Adults	0 / 888	100.00	99.57 - 100.00
	Children	0 / 264	100.00	98.57 - 100.00
	**Total**	0 / **1,152**	**100.00**	**99.67 - 100.00**

Binominal confidence intervals were computed using the Wilson score interval.

Supplementary Table 4. Assay sensitivity of anti-SARS-CoV-2 antibody detection assays in 332 serum samples from 99 COVID-19 patients collected between 0 and 16 weeks after the onset of symptoms.

Assay	Positve / Total	Sensitivity (%)	95% CI (%)
Architect Assay	86 / 98	87.76	79.81 - 92.85
EuroImmun Assay	85 / 99	85.86	77.65 - 91.39
Self-Developed Assay	74 / 97	76.29	66.93 - 83.65
recomLine Assay	88 / 97	90.72	83.30 - 95.04
Elecsys Assay	88 / 99	88.89	81.19 - 93.68

Binominal confidence intervals were computed using the Wilson score interval. The mean semi-quantitative results of all samples from the same patient was used to calculate the sensitivity, if more than one sample from the same patient was available.

Supplementary Table 5. Time resolved information on measures taken to prevent SARS-CoV-2 spread at the multicenter hospital until August 12, 2020.

Measures	Start date	End date
Prophylactic quarantine for travelers returning from risk areas	Jan 13 2020	-
rRT-PCR testing for all HCWs reporting high-risk exposures to SARS-CoV-2 infected individuals	Feb 28 2020	-
Voluntary rRT-PCR testing for HCWs reporting symptoms	Feb 28 2020	-
Prophylactic quarantine for non-essential HCWs reporting high-risk exposures to SARS-CoV-2 infected individuals	Feb 28 2020	-
Isolation of COVID-19 patients on specialised units	Mar 1 2020	-
Prohibition of business trips to risk areas for HCWs	Mar 6 2020	-
Cancellation and ban of meetings including larger groups of individuals	Mar 6 2020	Jun 6 2020
General visitation ban	Mar 17 2020	May 8 2020
No admission of patients for elective treatment	Mar 19 2020	Jun 7 2020
Face masks compulsory for all staff members	Mar 23 2020	-
Close-down of cafeterias and staff restaurants	Mar 28 2020	-
Face masks compulsory for patients during moving in the hospitals	Apr 6 2020	-
rRT-PCR testing for patients administerd to sugery upon admission	Apr 14 2020	-
Face mask compulsory for patients	Apr 15 2020	-
Allowance of one registered visitor for 1 h/day per patient	May 8 2020	May 29 2020
Allowance of several visitors per patient and day	May 29 2020	Jul 17 2020
rRT-PCR testing for all patients upon admission	Jun 4 2020	-
Allowance of one visitor for 1 h/day per patient	Jul 17 2020	-

Supplementary Table 6 (part 1). Anti-SARS-CoV-2 positivity rates of 7,554 health care workers from different departments/institutes and COVID-19 cases among staff members reported to the occupational health office.

Department/institute	Anti-SARS-CoV-2 Ab		Reported COVID-19 cases
	Pos. / total	%	
**conservative, internal medicine**	**53 / 1,157**	**4.58**	**82**
Department for Palliative Medicine†	1 / 67	1.49	0
Department of Infectious Diseases and Tropical Medicine*	0 / 51	0.00	0
Medical Clinic and Outpatient Clinic I*	11 / 230	4.78	19
Medical Clinic and Outpatient Clinic II*	4 / 123	3.25	12
Medical Clinic and Outpatient Clinic III*	18 / 307	5.86	18
Medical Clinic and Outpatient Clinic IV*	15 / 329	4.56	29
Medical Clinic and Outpatient Clinic V*	4 / 50	8.00	4
**conservative, non-internal medicine**	**45 / 1,911**	**2.35**	**22**
Central Emergency Department, Campus Großhadern*	1 / 54	1.85	4
Department for Aneasthesiology*	13 / 562	2.31	8
Department for Neurology and Friedrich Baur Institute*	7 / 231	3.03	4
Department for Nuclear Medicine†	3 / 81	3.70	2
Department for Psychiatry and Psychotherapy*	5 / 327	1.53	0
Department for Radiation Therapy and Radiation Oncology*	1 / 132	0.76	0
Department for Radiology*	5 / 217	2.30	0
Departmet for Dermatology and Allergology*	7 / 155	4.52	3
Institute for Clinical Neuroimmunology*	1 / 67	1.49	0
Institute for Diagnostical and Interventional Neuroradiology*	0 / 21	0.00	0
Institute for General Practice*	0 / 14	0.00	1
Institute of Occupational, Social and Environmental Medicine*	2 / 50	4.00	0
**surgical**	**30 / 1,952**	**1.54**	**20**
Department for General, Visceral, and Transplant Surgery*	1 / 230	0.43	1
Department for Gynecology and Obstetrics*	8 / 375	2.13	5
Department for Hand, Plastic, and Aesthetic Surgery*	0 / 30	0.00	0
Department for Heart Surgery*	5 / 115	4.35	1
Department for Neurosurgery*	1 / 137	0.73	2
Department for Ophthalmology*	2 / 154	1.30	1
Department for Oral and Maxillofacial Surgery*	1 / 58	1.72	3
Department for Orthopedics, Physical Medicine and Rehabilitation*	3 / 216	1.39	0
Department for Otorhinolaryngology*	3 / 121	2.48	1
Department for Thoracic Surgery*	1 / 24	4.17	0
Department for Trauma, and Reconstructive Surgery*	1 / 185	0.54	4
Department for Urology*	1 / 110	0.91	1
Department for Vascular Surgery*	0 / 24	0.00	0
Outpatient Clinic for Dental Prosthetics*	2 / 66	3.03	1
Outpatient Clinic for Orthodontics*	0 / 32	0.00	0
Outpatient Clinic for Tooth Preservation and Parodontology*	0 / 59	0.00	0
Outpatient Surgery Center*	1 / 16	6.25	0

†clinical departments/institutes that did not deploy personnel to COVID-19 units (non-COVID-19 response),
*clinical departments/institutes that deployed personnel to COVID-19 units (COVID-19 response)

Supplementary Table 6 (part 2). Anti-SARS-CoV-2 positivity rates of 7,554 health care workers from different departments/institutes and COVID-19 cases among staff members reported to the occupational health office.

Department/institute	Anti-SARS-CoV-2 Ab		Reported COVID-19 cases
	Pos. / total	%	
**pediatric**	**16 / 908**	**1.76**	**8**
Children's Palliative Center Munich†	1 / 39	2.56	0
Department for Child and Adolescent Psychiatry, Psychosomatics, and Psychotherapy*	4 / 121	3.31	1
Department for Pediatric Cardiology and Intensive Care*	1 / 61	1.64	0
Department for Pediatric Surgery, Dr. von Haunersches Kinderspital*	1 / 133	0.75	0
Department for Pediatrics, Dr. von Haunersches Kinderspital*	9 / 554	1.62	7
**Other departments with patient contact***	**6 / 207**	**2.90**	**0**
**non-clinical**	**15 / 1,419**	**1.06**	**14**
Accouting	0 / 36	0.00	0
Administrative Departments of the Board	1 / 60	1.67	0
Administrative Departments of the Commercial Management	0 / 42	0.00	0
Catering	0 / 40	0.00	1
Central Sterile Services	0 / 31	0.00	0
Department for Clinical Pharmacology	0 / 25	0.00	0
Department for Construction and Technical Facilities	1 / 108	0.93	0
Department for Medical Technology and IT	0 / 102	0.00	0
Department for Patient Logistics	0 / 22	0.00	0
Department for Patient Management	2 / 128	1.56	1
Department for Procurement and Economy	1 / 85	1.18	3
Department for Prophylaxis and Epidemiology of Cardiovascular Diseases	0 / 54	0.00	0
Department for Transfusion Medication, Cell Therapeutics and Hemostaseology	0 / 63	0.00	0
Dispensary	0 / 90	0.00	0
HR Department	1 / 54	1.85	1
Institute for Didactics and Medical Education Research	0 / 17	0.00	0
Institute for Human Genetics	0 / 11	0.00	0
Institute for Molecular Musculoskeletal Research	0 / 2	0.00	0
Institute for Psychiatric Phenomics and Genetics	0 / 7	0.00	0
Institute for Stroke and Dementia Research	2 / 116	1.72	1
Institute for Emergency Medicine and Medicine Management	0 / 33	0.00	0
Institute for Surgical Research	1 / 23	4.35	0
Institute for Laboratory Medicine	0 / 7	0.00	5
Occupational Health Office	0 / 2	0.00	0
other departments without patient contact	6 / 261	2.30	2

†clinical departments/institutes that did not deploy personnel to COVID-19 units (non-COVID-19 response),
*clinical departments/institutes that deployed personnel to COVID-19 units (COVID-19 response)
